# Hormone therapy use and duration with postoperative radiotherapy for recurrent prostate cancer: an individual patient data meta-analysis

**DOI:** 10.1016/S0140-6736(26)00137-6

**Published:** 2026-02-26

**Authors:** Amar U Kishan, Yilun Sun, Christopher C Parker, Paul Sargos, Matthew R Sydes, Sylvie Chabaud, Meryem Brihoum, Tahmineh Romero Kalbasi, Michael L Steinberg, Luca F Valle, Kekoa Taparra, Jonathan E Shoag, Jorge A Garcia, Jason R Brown, Matthew B Rettig, Adam E Singer, Robert E Reiter, Scott Eggener, Wayne Brisbane, Soumyajit Roy, Nicholas G Zaorsky, Angela Y Jia, Ting Martin Ma, Nicholas G Nickols, Jason A Efstathiou, Osama Mohamad, James J Dignam, Wendy F Seiferheld, Alan Pollack, Howard M Sandler, Paul L Nguyen, Pascal Pommier, Daniel E Spratt

**Affiliations:** Department of Radiation Oncology, University of California, Los Angeles, Los Angeles, CA, USA; Department of Radiation Oncology, University Hospitals Seidman Cancer Center, Case Western Reserve University School of Medicine, Cleveland, OH, USA; The Royal Marsden NHS Foundation Trust, London, UK; The Institute of Cancer Research, London, UK; Institut Bergonié, Bordeaux, France; MRC Clinical Trials Unit at UCL, Institute of Clinical Trials and Methodology, University College London, London, UK; Clinical Research Department, Centre Léon Bérard, Lyon, France; Unicancer, Paris, France; Department of Medicine Statistical Core, University of California, Los Angeles, Los Angeles, CA, USA; Department of Radiation Oncology, University of California, Los Angeles, Los Angeles, CA, USA; Department of Radiation Oncology, University of California, Los Angeles, Los Angeles, CA, USA; Department of Radiation Oncology, University of California, Los Angeles, Los Angeles, CA, USA; School of Public Health, University of California, Los Angeles, Los Angeles, CA, USA; Department of Urology, University Hospitals Seidman Cancer Center, Case Western Reserve University School of Medicine, Cleveland, OH, USA; Department of Medicine, University Hospitals Seidman Cancer Center, Case Western Reserve University School of Medicine, Cleveland, OH, USA; Department of Medicine, University Hospitals Seidman Cancer Center, Case Western Reserve University School of Medicine, Cleveland, OH, USA; Department of Medicine, University of California, Los Angeles, Los Angeles, CA, USA; Department of Medicine, University of California, Los Angeles, Los Angeles, CA, USA; Department of Urology, University of California, Los Angeles, Los Angeles, CA, USA; Department of Urology, University of California, Los Angeles, Los Angeles, CA, USA; Department of Urology, University of California, Los Angeles, Los Angeles, CA, USA; Department of Radiation Oncology, University Hospitals Seidman Cancer Center, Case Western Reserve University School of Medicine, Cleveland, OH, USA; Department of Radiation Oncology, University Hospitals Seidman Cancer Center, Case Western Reserve University School of Medicine, Cleveland, OH, USA; Department of Radiation Oncology, University Hospitals Seidman Cancer Center, Case Western Reserve University School of Medicine, Cleveland, OH, USA; Department of Radiation Oncology, University of Washington, Seattle, WA, USA; Department of Radiation Oncology, University of California, Los Angeles, Los Angeles, CA, USA; Department of Radiation Oncology, Greater Los Angeles VA, Los Angeles, CA, USA; Department of Radiation Oncology, Massachusetts General Hospital, Harvard Medical School, Boston, MA, USA; Department of Radiation Oncology, MD Anderson Cancer Center, Houston, TX, USA; Department of American University of Beirut, Beirut, Lebanon; Department of Public Health Sciences, University of Chicago, Chicago, IL, USA; NRG Oncology Statistics and Data Management Center, Philadelphia, PA; NRG Oncology Statistics and Data Management Center, Philadelphia, PA; Department of Radiation Oncology, Miller School of Medicine and Sylvester Comprehensive Cancer Center, University of Miami, Miami, FL, USA; Department of Radiation Oncology, Cedars-Sinai Medical Center, Los Angeles, CA, USA; Department of Radiation Oncology, Brigham and Women’s Hospital and Dana Farber Cancer Institute, Boston, MA, USA; Clinical Research Department, Centre Léon Bérard, Lyon, France; Department of Radiotherapy, Institut Curie, Paris, France; Department of Radiation Oncology, University Hospitals Seidman Cancer Center, Case Western Reserve University School of Medicine, Cleveland, OH, USA

## Abstract

**Background:**

Adding hormone therapy to definitive radiotherapy in localised prostate cancer improves overall survival, but whether it similarly improves overall survival in the context of postoperative radiotherapy (PORT) after radical prostatectomy is unclear. Herein, we report an individual patient data (IPD) meta-analysis of randomised trials aimed at quantifying the benefit of adding hormonal therapy to PORT.

**Methods:**

This was an IPD meta-analysis that identified randomised, phase 3 trials of PORT with or without hormone therapy. A systematic literature search of MEDLINE, Embase, trial registries, the Web of Science, Scopus, and relevant conference proceedings was done on Dec 15, 2024. IPD were available via the MARCAP consortium. The primary outcome was overall survival. Meta-analyses evaluated the benefit of adding hormone therapy, short-term hormone therapy (4–6 months), or long-term hormone therapy (24 months) to PORT. Tests for interaction based on pre-PORT prostate-specific antigen (PSA) and duration of hormone therapy were evaluated and non-linear associations between pre-PORT PSA and overall survival were modelled. This study was done under the master protocol of the MARCAP Consortium (PROSPERO registration CRD42019134376).

**Findings:**

IPD were available for six randomised trials including 6057 patients with a median follow-up of 9·0 years (IQR 7·2–10·7 years). Adding hormone therapy to radiotherapy did not significantly improve overall survival (hazard ratio [HR] 0·87, 95% CI 0·76–1·01, p=0·06). There was no significant interaction between hormone therapy duration and this effect (p_interaction_=0·17), although there was a significant interaction with pre-PORT PSA greater than 0·5 ng/mL versus 0·5 ng/mL or less (p_interaction_=0·02). For all pre-PORT PSA values, the upper bounds of the 95% CI of the HR for overall survival crossed 1·0 for patients randomly assigned to PORT with or without short-term hormone therapy (n=3938). For patients randomly assigned to PORT with or without long-term hormone therapy (n=1088), the upper bounds of the 95% CI for overall survival HR fell below 1·0 at PSA greater than 1·6 ng/mL.

**Interpretation:**

Our findings, we believe, provide the strongest level of evidence to date suggesting there might be no meaningful overall survival benefit to adding hormone therapy, either short-term or long-term hormone therapy, to PORT for PSA 0·5 ng/mL or less, with no apparent difference in efficacy for short-term versus long-term hormone therapy. There is an unmet need to identify biomarkers to predict potential hormone therapy benefit.

**Funding:**

National Institutes of Health.

## Introduction

Definitive radiotherapy is a standard treatment option for men with localised prostate cancer. In patients with intermediate-risk and high-risk prostate cancer, the addition of short-term hormone therapy (4–6 months) to definitive radiotherapy and the adjuvant prolongation of short-term hormone therapy to long-term hormone therapy (18–36 months) have been shown to improve overall survival.^1^

Recurrence after radical prostatectomy, the surgical removal of the prostate and seminal vesicles as a definitive treatment of prostate cancer, can occur, often necessitating postoperative radiotherapy (PORT).^2^ Given the benefit of hormone therapy with definitive radiotherapy, several randomised trials investigated the benefit of adding short-term hormone therapy or long-term hormone therapy to PORT,^3–8^ but to date only one has shown an overall survival benefit.^7^ The lack of an overall survival benefit to adding hormone therapy to PORT was supported by the results of a recent aggregate study-level meta-analysis of these trials.^9,10^ However, owing to potential heterogeneity in absolute and relative treatment effects in this diverse population, individual patient data are requisite to assess with more granularity any covariable and subgroup effects on overall survival.

Leveraging the Meta-Analysis of Randomised trials in Cancer of the Prostate (MARCAP) Consortium, which has allowed a rigorous and detailed evaluation of the benefit of hormone therapy for definitive radiotherapy,^1^ we sought to do an individual patient data meta-analysis (Post Operative Salvage or Adjuvant Radiotherapy and anti-androgen therapy for men with prostate cancer; POSEIDON) of all published randomised, phase 3 trials of PORT with or without hormone therapy. We hypothesised that the addition of hormone therapy to postoperative radiotherapy would not improve overall survival, but the benefit might be modulated on the basis of pre-PORT PSA levels.

## Methods

### Search strategy and selection criteria

To identify all relevant randomised, phase 3 trials investigating the addition of hormone therapy to PORT, or the prolongation from short-term to long-term hormone therapy with PORT, we did a systematic literature review. This study was reported in accordance with the PRISMA guidelines.^11^ Our statistical analysis plan and a completed checklist is provided in the appendix (pp 3–8).^12^ A systematic literature search was done in the English language by use of MEDLINE (1966–2025), Embase (1982–2025), trial registries (Cochrane Central Register of Controlled Trials and ClinicalTrials.gov), the Web of Science, Scopus, and major urology and oncology conference proceedings (1990–2025) to retrieve relevant studies. Controlled vocabulary was leveraged for studies involving humans by use of the following terms: “randomised” AND “prostate” AND (“androgen deprivation” OR “hormone therapy”) AND (“radiotherapy” OR radiation”) AND (“surgery” OR “prostatectomy”). The search was done on Dec 15, 2024. We had two prespecified exclusion criteria: trials that did not collect distant metastasis or survival data or were single centre. The search was done by a single investigator (AUK) and verified by another (DES). Our search identified six trials eligible for analysis (figure 1 and appendix pp 10–12).^3–8^ All six trials were available through the MARCAP consortium and were included in this meta-analysis.^3–8^

The MARCAP consortium has been described in detail previously.^1^ Briefly, it contains individual patient data from randomised, clinical trials run through multiple collaborative groups. The requirement for ethics approval was waived by the University of California Los Angeles and University Hospitals Cleveland Medical Center institutional review boards.

### Data analysis

Data extraction and preparation for each individual trial were done by the relevant governing body. The data requested included patient age, pathological T category, pathological Gleason score, PSA before radiotherapy, surgical margin status, presence of seminal vesicle invasion, presence of extracapsular extension, and allocated treatment. Outcomes data requested included time to event information and event status for biochemical, local, regional, and distant failure, death, and cause of death. Updated survival status and time to last follow-up were requested from the trialist groups. Data were shared with the coprincipal investigators (AUK and DES) and statisticians (YS and TRK) of the MARCAP consortium (secured at UH Seidman Cancer Center, Cleveland, OH, and UCLA, Los Angeles, CA, USA). Data underwent intensive harmonisation and integrity checks consistent with best-practice frameworks, including external consistency checks, whereby data were cross-referenced against primary publications to replicate baseline characteristics and primary outcome results; internal consistency and plausibility checks for logical date sequencing, variable ranges, and missing data patterns; and assessment of randomisation balance.^13^ No noteworthy integrity issues were identified.

The primary meta-analysis focused on the effect of adding hormone therapy to radiotherapy, irrespective of hormone therapy duration, versus radiotherapy alone. A test for interaction of the treatment effects and overall survival was done between trials that used short-term hormone therapy (ie, 4–6 months) or long-term hormone therapy (ie, 24 months), as well as subset meta-analyses within each treatment subgroup. A specific exploratory analysis comparing short-term hormone therapy versus long-term hormone therapy with PORT was done, restricted to the subset of trials that directly randomly assigned patients to these distinct durations (RADICALS-HD).^5,8^ For the short-term hormone therapy meta-analysis, the two groups in NRG/RTOG 0534 that included hormone therapy were combined for comparison against the PORT alone group.^6^ Metrics for between-trial heterogeneity were calculated and summarised in the appendix (p 13).

### Outcomes

The primary objective of this study was to establish the impact of treatment intensification on overall survival. The secondary objectives were to assess the impact of each treatment intensification method on rates of metastasis-free survival (MFS), event-free survival, and biochemical recurrence. For all endpoints, time-to-event was calculated from the date of randomisation to the date of the first qualifying event, or censored at the date of last follow-up. Event-free survival was defined as the composite time to biochemical recurrence, clinical progression, initiation of salvage treatment, or death from any cause. Although biochemical recurrence definitions varied across the included eras, they were consistent within each trial; therefore, to account for this heterogeneity, biochemical recurrence was analysed strictly within the context of the trial-stratified models described below, with death from any cause as a competing risk.

### Statistical analysis

Outcomes and analyses were defined a priori in a study-specific statistical analysis plan. This plan was discussed and finalised by the co-principal investigators and statisticians before data harmonisation and analysis. This study was done under the master protocol of the MARCAP Consortium (PROSPERO registration CRD42019134376). The full statistical analysis plan, including the prespecified hypotheses, subgroup categorisations, and methodology, is available in the appendix (pp 3–4).

Median follow-up time and IQR were calculated by use of the reverse Kaplan–Meier method. Between-trial heterogeneity was assessed by use of a two-stage random-effects meta-analysis based on the generic inverse variance method. Heterogeneity was quantified by use of τ^2^ (estimated via REML), *I*^2^, and Cochran’s Q, alongside prediction intervals.

The primary efficacy analysis used a one-stage meta-analysis framework. Data from all patients were analysed by use of a univariable Cox proportional hazards model (for overall survival, MFS, and event-free survival) or a univariable Fine–Gray subdistribution hazard model (for biochemical recurrence) assuming a common treatment effect, stratified by trial. This stratification allows the baseline hazard function to vary between trials, thereby absorbing heterogeneity in event definitions, follow-up duration, and era-specific risks; treatment effects were thus estimated by comparing patients strictly with concurrent controls within the same trial stratum. Notably, the original RADICALS-HD short versus none^4^ and RADICALS-HD long versus short^8^ publications included patients who were randomly assigned in both the respective two-way cohorts (short *vs* none and long *vs* short 1:1 randomisations) as well as patients randomly assigned in the RADICALS-HD 1:1:1 three-way randomisation.^5^ For the present POSEIDON meta-analysis, the availability of individual patient data allowed the ability to treat the randomisations as completely distinct trials (ie, patients were not double-counted).^14^ For the short-term hormone therapy use meta-analysis, across trials of PORT with or without short-term hormone therapy, all patients receiving PORT alone were pooled together to form one group and those receiving PORT with short-term hormone therapy were pooled together to form the comparator group. Four trials contributed to the PORT with or without short-term hormone therapy meta-analysis, all of which predominantly used gonadotropin releasing hormone agonist-based hormone therapy; details on the included trials are shown in the appendix (pp 10–12). Two trials contributed to the PORT with or without long-term hormone therapy meta-analysis: RADICALS three-way, which used 24 months of androgen deprivation therapy with gonadotropin releasing hormone agonist medications for most patients (425 [86%] of 492 patients) and also included a third randomised group evaluating 6 months of hormone therapy plus radiotherapy (with the no-hormone therapy group serving as the common control),^5^ and RTOG 9601, which used high dose non-steroidal androgen receptor antagonist monotherapy with bicalutamide.^7^

Overall survival and MFS were reported by use of the HR with 95% CIs from stratified univariable Cox regression models along with absolute differences in Kaplan–Meier event probability estimates at 10 years.^15,16^ The Grambsch–Therneau test was applied to identify violation of the proportionality assumption of the Cox regression models. For each treatment group, 10-year restricted mean survival time and 10-year restricted mean time lost were calculated to supplement relative risks. Restricted mean survival time was established from the Kaplan–Meier estimate of the survival function, whereas restricted mean time lost was established from the estimated cumulative incidence function.

Subgroup analyses were done within strata defined by randomisation to with or without short-term hormone therapy versus with or without long-term hormone therapy, age (<60 years *vs* 60–64 years *vs* 65–69 years *vs* >70 years), pathological Gleason score (6 *vs* 7 *vs* 8–10), pre-PORT PSA (≤0·20 ng/mL *vs* 0·21–0·50 ng/mL *vs* 0·51–1·00 ng/mL *vs* >1·00 ng/mL), surgical margin status (negative *vs* positive), seminal vesicle invasion (yes *vs* no), and extracapsular extension (yes *vs* no) with formal testing for interaction effects by use of separate multivariable models for each covariate of interest. Each model included the treatment, the specific covariate, and a treatment-by-covariate interaction term. For these models, PSA was analysed as both a continuous variable and as a categorical variable with the aforementioned four strata. For categorical and ordinal subgroups, we used a global test of interaction (simultaneous test of effects) to detect any heterogeneity in treatment effect across strata without assuming a linear trend. As a sensitivity analysis to assess potential aggregation bias, we also did two-stage meta-analyses of within-trial interaction effects. Interaction coefficients and their corresponding variance–covariance matrices were estimated within each trial independently and subsequently pooled by use of random-effects models to strictly isolate within-trial treatment-subgroup contrasts.

On the basis of previous work suggesting that pre-PORT PSA is both a prognostic and a potentially predictive biomarker for the benefit of long-term hormone therapy in this context,^17^ the effect of pre-PORT PSA was explored further. First, to assess for any interaction between pre-PORT PSA and the effect of hormone therapy on MFS and overall survival, multivariable models adjusted for age, pathologic Gleason score, and pathological T stage were done on the basis of PSA as a continuous variable, PSA with the aforementioned categorical separation (≤0·20 ng/mL *vs* 0·21–0·50 ng/mL *vs* 0·51–1·00 ng/mL *vs* >1·00 ng/mL), and PSA with a binary categorisation (≤0·5 ng/mL *vs* >0·5 ng/mL). Second, absolute risk reductions at 10 years for distant-metastasis-or-all-cause-mortality events (as a composite endpoint) and for all-cause mortality events were calculated as the differences in event risks, estimated with the Kaplan–Meier method at 10 years between the treatment groups, correspondingly. The numbers-needed-to-treat (NNTs) for these same endpoints for the addition of hormone therapy to radiotherapy, and separately for the addition of short-term hormone therapy and long-term hormone therapy, were calculated as the inverse of the absolute risk reduction at 10 years. Third, natural cubic splines modelling non-linear associations between pre-PORT PSA and overall survival and MFS were developed for the addition of hormone therapy to radiotherapy, by use of stratified Cox models. The number of knots was selected from grid search ranging from 1 to 5 on the basis of the lowest Akaike Information Criterion. Splines were adjusted for pathological Gleason score, age, and pathological T stage. A previous meta-analysis had suggested a surrogate threshold effect for MFS on overall survival in localised prostate cancer, such that interventions for which the upper 95% CI of the hazard ratio (HR) for MFS falls below 0·81 would be expected to show a significant benefit in overall survival.^18^ Missing data for baseline covariates were minimal (<maximum 218 of 6057 or 3·6% for Gleason score) and balanced between treatment groups. Consequently, analyses involving covariates were done by use of a complete-case analysis. Finally, we did not apply corrections for multiple testing owing to the high correlation between outcomes and the prespecified nature of our interaction analyses; therefore, secondary subgroup findings should be interpreted as hypothesis-generating. All analyses were done on an intention-to-treat basis by use of R (version 4.3.2) at a two-tailed significance level of 0·05.

### Role of the funding source

The funders of the study had no role in study design, data collection, data analysis, data interpretation, or writing of the report.

## Results

Overall, data were available for six randomised trials including 6057 patients with a median follow-up of 9·0 years (IQR 7·2–10·7), of whom 5026 contributed to the primary comparison of PORT with versus without hormone therapy. No data integrity issues were identified. Details on the included trials are shown in the appendix (pp 10–12, 18). Patient characteristics are shown in table and the appendix (pp 14–15) and were well-balanced within each meta-analysis. Trial-specific estimates are reported in the appendix for the addition of any hormone therapy (appendix p 19), addition of short-term hormone therapy (appendix p 22), addition of long-term hormone therapy (appendix p 24), and prolongation of short-term therapy to long-term therapy (appendix p 26). Race and ethnicity data were not available.

Six randomised comparisons across five randomised trials evaluated the addition of hormone therapy to PORT (5026 patients, median follow-up of 9·10 years [IQR 7·30–10·97]; table). Survival curves for the addition of hormone therapy to PORT in the overall population, as well as in subgroups based on pre-PORT values of up to 0·20 ng/mL, 0·21–0·50 ng/mL, 0·51–1·00 ng/mL, and greater than 1·00 ng/mL are shown in figure 2. There was no clear evidence that the addition of hormone therapy to radiotherapy improved overall survival (HR 0·87, 95% CI 0·76–1·01, p=0·06), with 10-year overall survival rates of 83·6% (95% CI 81·8–85·4%) with PORT alone versus 84·3% (95% CI 82·7–86·0) with the addition of hormone therapy. For patients with PSA up to 0·2 ng/mL (HR 1·14, 95% CI 0·83–1·57) and PSA 0·21–0·50 ng/mL (HR 0·94, 0·74–1·19), HRs did not indicate an overall survival benefit from hormone therapy. In contrast, patients with PSA 0·51–1·00 ng/mL (HR 0·72, 95% CI 0·54–0·96) and PSA >1·00 ng/mL (HR 0·69, 0·48–0·98) showed a reduction in mortality risk from the addition of hormone therapy (nominal p<0·05), consistent with the significant interaction observed in the binary analysis. On univariable analysis, the interaction term based on categorical binning pre-PORT PSA into these four strata was not significant (p_interaction_=0·08). Using a two-stage multivariate meta-analysis to strictly isolate within-trial contrasts and mitigate potential aggregation bias, we confirmed a significant interaction between the addition of hormone therapy and pre-PORT PSA. Notably, when PSA was analysed as a continuous variable, the interaction for overall survival was significant in the two-stage model (p=0·04), whereas the one-stage stratified model had previously yielded a non-significant result (p=0·19). The binary interaction at a PSA threshold of 0·5 ng/mL also remained robust in the two-stage framework (p=0·03).

For patients randomly assigned to with or without long-term hormone therapy, the hazard ratio for overall survival favoured the addition of hormone therapy (HR 0·79, 95% CI 0·63–1·00), whereas those randomly assigned to with or without short-term hormone therapy did not (HR 0·93, 0·77–1·11; figure 3). However, the interaction p value between randomisation to with or without short-term hormone therapy versus with or without long-term hormone therapy was not significant (p_interaction_=0·17). Results were similar on multivariable analysis. Notably, patients enrolled in with or without long-term hormone therapy trials had higher pre-PORT PSAs and more adverse pathological features than those enrolled in with or without short-term hormone therapy trials.

The NNTs to prevent one death at 10 years by adding hormone therapy to radiotherapy were negative for PSA values between 0·0 and 0·5 ng/mL, reflecting point estimates that numerically favoured PORT alone; these estimates are imprecise and should be interpreted cautiously. For patients with pre-PORT PSAs of 0·51–1·00 ng/mL and greater than 1·00 ng/mL, the NNTs were 22 and 12, respectively. Adjusted cubic splines evaluating non-linear associations between pre-PORT PSA concentration and overall survival and MFS are shown in figure 4A–B. For both endpoints, the upper bounds of the 95% CI for the HR cross 1·0 (ie, ln[HR] crosses 0) for all pre-PORT PSAs. Consistent with this, for MFS the Intermediate Clinical Endpoints in Cancer of the Prostate (ICECaP) surrogate threshold effect of 0·81 was crossed by the upper bounds of the 95% CI (ie, upper bounds of ln[HR] crossed −0·21) for all pre-PORT PSAs.

The addition of hormone therapy was associated with improved MFS (HR 0·79, 95% CI 0·70–0·89, p<0·001), with 10-year MFS rates of 77·9% (95% CI 76·0–79·8%) versus 74·1% (72·0–76·3%) with the addition of hormone therapy (figure 2F). On univariable analysis, the interaction term based on binning pre-PORT PSA into the aforementioned four strata was not significant (p_interaction_=0·1; appendix pp 20–21). Results were similar on multivariable analysis or with binary PSA binning or treating PSA as a continuous variable. NNTs to prevent one death-or-metastasis event at 10 years were negative for pre-PORT PSA values of up to 0·2 ng/mL, and were 21 for PSA for pre-PORT PSA values of 0·21–0·50 ng/mL, 14 for those of 0·51–1·00 ng/mL, and seven for those of greater than 1·00 ng/mL (appendix p 16).

Four randomised trials evaluated the addition of short-term hormone therapy to radiotherapy (3938 patients, median follow-up 8·80 years [IQR 7·13–10·10]; table). On spline analysis, the upper bounds of the 95% CI for the HR crossed 1·0 for overall survival and MFS endpoints, as well as crossing the surrogate threshold effect for MFS (figure 4C–D), for all pre-PORT PSAs. The addition of short-term hormone therapy to radiotherapy did not improve overall survival (HR 0·93, 95% CI 0·77–1·11; figure 5A), but improved MFS (HR 0·82, 95% CI 0·71–0·95; figure 5B).

The NNTs to prevent one death at 10 years by adding short-term hormone therapy to radiotherapy were negative for PSA values between 0·0 and 0·5 ng/mL, again reflecting point estimates that numerically favoured PORT alone and should be interpreted cautiously, and were 18 for patients with pre-PORT PSAs of 0·51–1·00 ng/mL and 12 for patients with pre-PORT PSAs greater than 1·00 ng/mL. NNTs to prevent death-or-metastasis events are shown in the appendix (p 16). Subgroup analyses are shown in the appendix (p 23).

Two randomised trials evaluated adding long-term hormone therapy to PORT (1088 patients, median follow-up 12·34 years [IQR 9·37–13·86]; table). Both trials included a comparison of 24 months of hormone therapy with PORT versus PORT alone. The addition of long-term hormone therapy to PORT was associated with improved overall survival (HR 0·79, 95% CI 0·63–1·00, p=0·049) and MFS (HR 0·74, 95% CI 0·60–0·91, p=0·004), corresponding to 10-year overall survival estimates of 82·7% (79·3–86·1) versus 79·8% (76·2–83·4; figure 5C–D).

The NNTs to prevent one death at 10 years by adding long-term hormone therapy to radiotherapy were negative for PSA values of 0·2 ng/mL or less, indicating point estimates that numerically favoured PORT alone but with considerable uncertainty, and were 45 for patients with pre-PORT PSAs of 0·21–0·50 ng/mL, 16 for patients with pre-PORT PSAs of 0·51–1·00 ng/mL, and 12 for patients with pre-PORT PSAs greater than 1·00 ng/mL. NNTs for death-or-metastasis events are shown in the appendix (p 16). Subgroup analyses are shown in the appendix (p 25). On spline analysis, the upper bounds of the 95% CI for the HR crossed 1·0 for both endpoints: at PSAs of at least 1·6 ng/mL for overall survival and at least 1·2 ng/mL for MFS (figure 4E–F). The ICECaP threshold for the MFS effect having a subsequent effect on overall survival was met for PSA values of at least 2·0 ng/mL (figure 4F).

An exploratory analysis evaluated the benefit of prolonging short-term hormone therapy to long-term hormone therapy with PORT; this analysis used the two-way and three-way RADICALS trials and included 1523 patients with a median follow-up of 8·89 years (IQR 7·03–10·05; appendix pp 14–15). The prolongation of hormone therapy did not improve overall survival (HR 0·89, 95% CI 0·68–1·16, p=0·37), but did improve MFS (HR 0·76, 95% CI 0·61–0·95, p=0·02; figure 5E–F). Subgroup analyses and estimated NNTs for death and death-or-metastasis are shown in the appendix (pp 16, 27).

## Discussion

To our knowledge, this is the first individual patient data meta-analysis of randomised trials evaluating the effect of adding hormone therapy to PORT in prostate cancer, with several immediately clinically relevant results. First, the addition of hormone therapy to PORT does not clearly provide relative benefits in overall survival, particularly for patients receiving radiotherapy at pre-PORT PSA values of 0·5 ng/mL or less, with a small absolute benefit. Second, the treatment effects observed from short-term and long-term hormone therapy appear more similar than different, with a possible exception in men with pre-PORT PSA levels greater than 1·6 ng/mL. Finally, although adding hormone therapy does appear to improve MFS, the magnitude of this benefit was small, implying a small effect on overall survival until pre-PORT PSA levels exceed 2·0 ng/mL. Thus, overall, the POSEIDON results do not support the routine addition of hormone therapy to PORT for patients receiving adjuvant or early secondary (previously called salvage radiotherapy) and suggests that meaningful benefits in overall survival would primarily manifest for patients receiving very late secondary radiotherapy (PSA>1·6–2·0 ng/mL).

Our results complement the recent study-level DADSPORT meta-analysis that evaluated the effect of adding hormone therapy to PORT. We show similar effect sizes for overall survival and MFS with the addition of hormone therapy to PORT.^10^ However, the DADSPORT authors explicitly noted that “collection and reanalysis of individual patient data may help clarify who might benefit”.^10^ The availability of such data for POSEIDON addressed this need by enabling granular analysis of the non-linear interaction of pre-PORT PSA with treatment effect, identifying potential interaction thresholds missed by aggregate trend tests, and standardising covariate definitions across trials.

Specifically, we present important analyses of non-linear associations between pre-PORT PSA and the effect of adding hormone therapy. For both the overall cohort and for men in trials evaluating PORT with or without hormone therapy, the upper bounds of the 95% CI for the HR crossed 1·0 for all evaluated PSA levels for both overall survival and MFS. For MFS, the upper bounds of the 95% CI for the HR not only always crossed 1·0 but also always crossed 0·81, the surrogate threshold effect limit defined by ICECaP for an MFS effect likely to translate into a subsequent overall survival effect.^18^ Therefore, the observed MFS benefits would not be expected to translate to overall survival benefits. For long-term hormone therapy specifically, the upper bounds of the 95% CI for the overall survival HR crossed 1·0 for pre-PORT PSAs of at least 1·6 ng/mL, and for MFS, the ICECaP threshold was passed for pre-PORT PSAs of at least 2·1 ng/mL. This suggests that long-term hormone therapy might provide survival benefits only when treating at very high pre-PORT PSA levels. This group of patients might also derive benefit from an intermittent hormone therapy approach that includes second generation androgen receptor antagonists.^20^ The NNT analysis also underscores caution with use of hormone therapy at lower PSA levels: for pre-PORT PSA of 0·5 ng/mL or lower, the NNTs to prevent one death at 10 years with adding either hormone therapy or short-term hormone therapy are less than 0, suggesting that, on average, the addition of hormone therapy or short-term hormone therapy might be harmful. In contrast, at pre-PORT PSA >0·5 ng/mL, the NNTs to prevent death at 10 years are 22 with the addition of hormone therapy, indicative of an absolute overall survival benefit of ≥5%. Taken together, these data suggest that for patients receiving early secondary radiotherapy (pre-PORT PSA ≤0·5 ng/mL), there is generally no survival advantage to adding hormone therapy, with benefits emerging only for pre-PORT PSA greater than 0·5 ng/mL.

One might be tempted to conclude that the addition of long-term hormone therapy provided greater benefit than short-term hormone therapy given that the 95% CI for overall survival did not cross 1·0 for long-term hormone therapy, but did for short-term hormone therapy. However, there was no evidence of an interaction on the basis of hormone therapy duration, nor was there an overall survival benefit on meta-analysis comparing short-term versus long-term hormone therapy. The observed results are probably the result of the overall higher preponderance of adverse risk features and higher pre-PORT PSA levels for patients enrolled in trials of PORT with or without long-term hormone therapy. Thus, short-term hormone therapy, if hormone therapy is used, appears to result in similar overall survival to long-term hormone therapy on the basis of the available randomised data.

The results from POSEIDON have notable contrast with the findings pertaining to the benefit of adding hormone therapy with definitive radiotherapy for prostate cancer.^1^ There are several potential explanations for this finding. Hormone therapy is known to be a potent radiosensitiser and, in the definitive management of prostate cancer with radiotherapy, improves overall survival.^21,22^ In the postoperative setting, the addition of hormone therapy might not meaningfully improve local control, and does not clearly offer a meaningful survival benefit. This hypothesis is supported by two negative randomised trials of dose-escalation in postoperative radiotherapy.^23,24^ Alternatively, the benefit of adding hormone therapy to PORT at high PSA levels in the postoperative setting could be explained by the treatment (albeit temporarily) of occult metastatic disease given the established correlation of metastatic disease on prostate-specific membrane antigen positron emission tomography (PSMA PET–CT) with pre-PORT PSA.

This meta-analysis is not without limitations. First, the analysis includes patients enrolled in trials over a 17-year period (1998–2015) during which definitions of biochemical recurrence varied, and both stage migration and Gleason grade migration occurred.^25,26^ However, our use of models stratified by trial ensures that comparisons are made strictly against concurrent controls subject to the same definitions, and given the consistency of the trial-specific results and the lack of significant heterogeneity, this is unlikely to have substantially impacted our findings. Second, the majority of patients in the analysis received hormone therapy in the form of androgen deprivation therapy delivered via gonadotropin releasing hormone agonist medications; the major exception was the use of androgen receptor antagonist monotherapy in NRG/RTOG 9601,^7^ which contributed 70% of the patients (760/1088 patients) in the radiotherapy with or without hormone therapy subgroup. Since androgen receptor antagonist monotherapy is less effective than traditional androgen deprivation therapy,^27^ this might be expected to dilute or obscure the results. However, tests for heterogeneity were non-significant. Molecular imaging, such as PSMA PET–CT, has greatly improved sensitivity for identifying occult disease at the time of post-prostatectomy recurrence.^28,29^ These scans were not generally available during the time interval in which the trials included in the POSEIDON analysis were done. In addition, detailed information on baseline comorbidities, PSA doubling times, and testosterone recovery, which might affect outcomes, was not available.^30^ Although our analysis was designed to focus on overall survival primarily, and MFS secondarily, earlier endpoints such as biochemical recurrence might be of great impact for patients. These endpoints, however, are inherently more variable between trials, which had varying definitions of biochemical recurrence and varying protocols for follow-up. Additionally, a larger impact on overall survival with longer follow-up cannot be ruled out. Further, in the RADICALS-HD randomisations,^4,5,8^ more patients in the with or without hormone therapy randomisation had secondary radiotherapy versus adjuvant radiotherapy than in the three-way randomisation, probably owing to different perceived risks before enrolment. Finally, in our primary assessment of interactions, we used standard one-stage models that do not explicitly separate within-trial from between-trial interaction effects. To address concerns regarding potential aggregation bias, we did sensitivity analyses using a two-stage approach that isolates within-trial interactions. The key finding that the significant interaction between pre-PORT PSA and treatment effect on overall survival was confirmed in the two-stage analysis (binary PSA p=0·03 *vs* p=0·02 in one-stage), and overall results were consistent across approaches (appendix pp 18–19).

In summary, the POSEIDON meta-analysis from the MARCAP consortium is, to our knowledge, the first individual patient data meta-analysis of randomised trials evaluating the use and prolongation of hormone therapy with postoperative radiotherapy in prostate cancer. We provide what we believe to be the strongest level of evidence to date suggesting that the addition of hormone therapy to postoperative radiotherapy might not benefit men receiving early salvage radiotherapy at pre-PORT PSA of 0·5 ng/mL or less. Although robust subgroup-specific benefits could not be broadly identified, pre-PORT PSA appears to be integral, with patients who have particularly elevated pre-PORT PSA levels (>1·6–2·0 ng/mL) appearing to derive the greatest potential benefits from the addition of hormone therapy. Notably, in this group of patients, adding short-term hormone therapy might be as beneficial as adding long-term hormone therapy. Predictive biomarkers are urgently needed to improve hormone therapy personalisation. NRG GU006 (NCT03371719) aims to address this unmet need by testing whether a transcriptionally defined biomarker, PAM50, can identify a differential treatment effect of adding short-term hormone therapy with apalutamide to PORT.

## Supplementary Material

Appendix (M 18480)

## Figures and Tables

**Figure 1:**
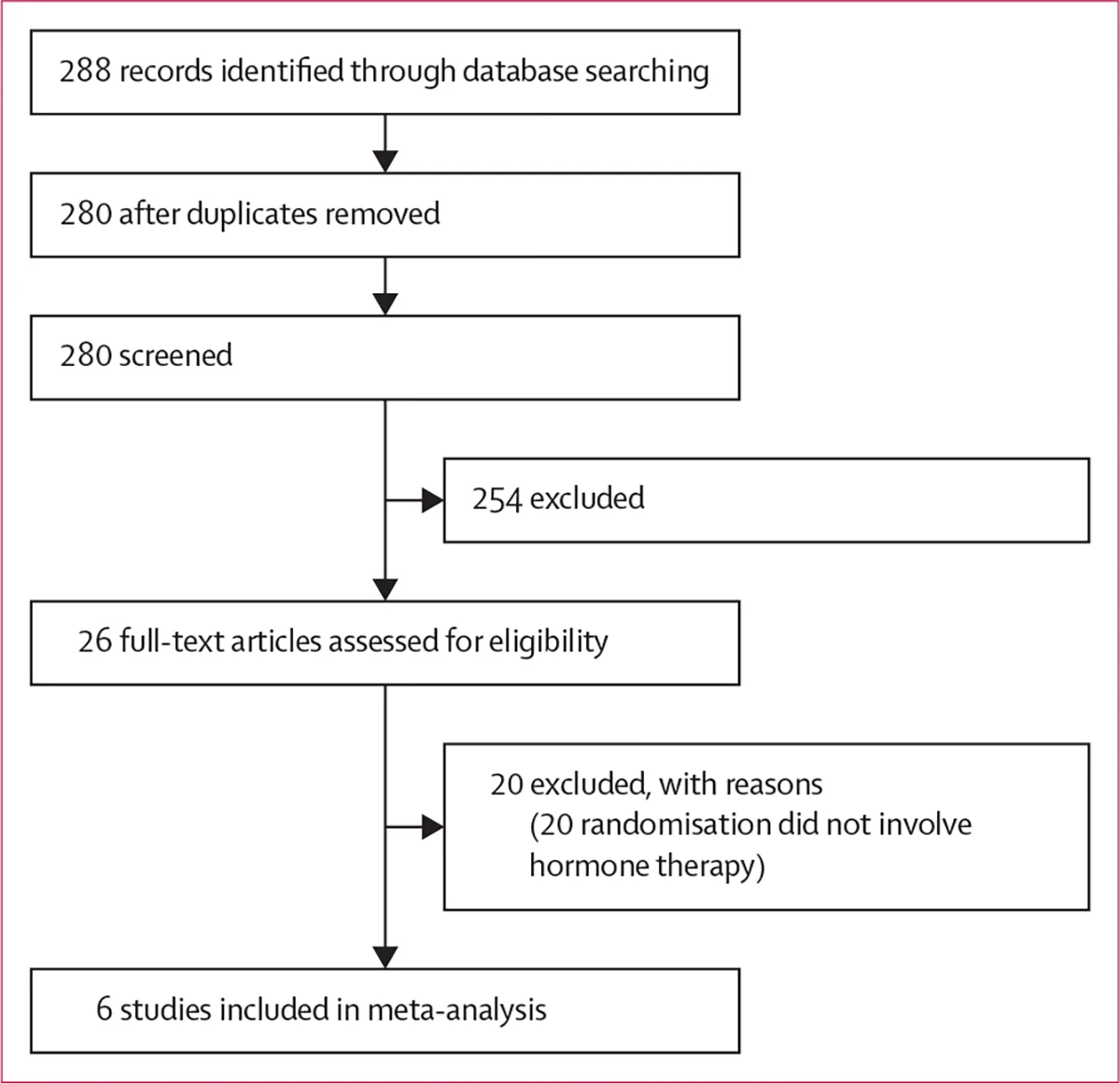
Trial profile

**Figure 2:**
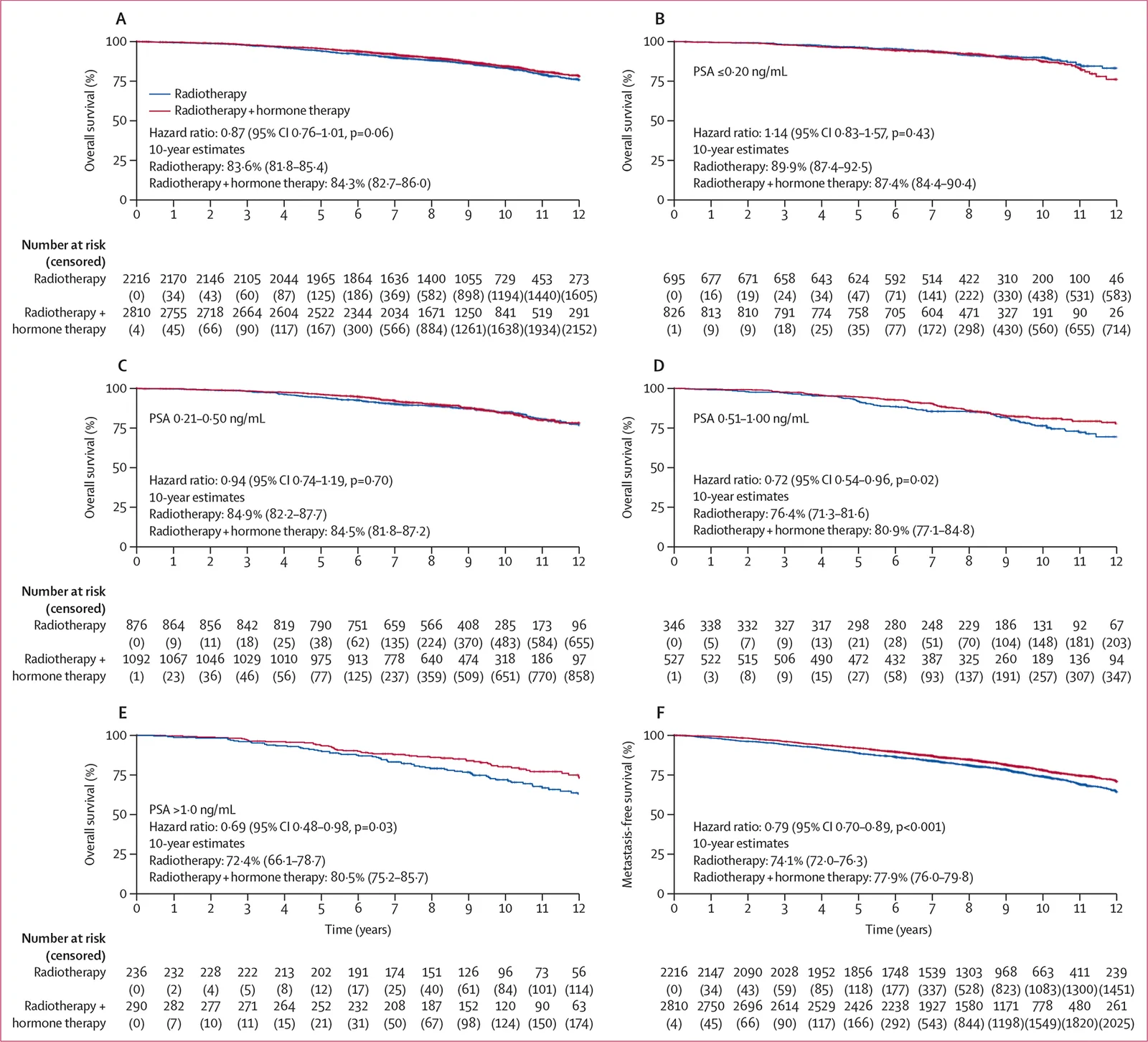
Survival effect of adding hormonal therapy to post-prostatectomy radiotherapy Kaplan–Meier survival curves showing overall survival impact of adding hormonal therapy to post-prostatectomy radiotherapy In the overall cohort (A) and in patients with pre-PORT PSA ≤0·20 ng/mL (B), 0·21–0·50 ng/mL (C), 0·51–1·00 ng/mL (D), and >1·00 ng/mL (E). (F) Kaplan–Meier survival curve for metastasis-free survival. PSA=prostate-specific antigen.

**Figure 3:**
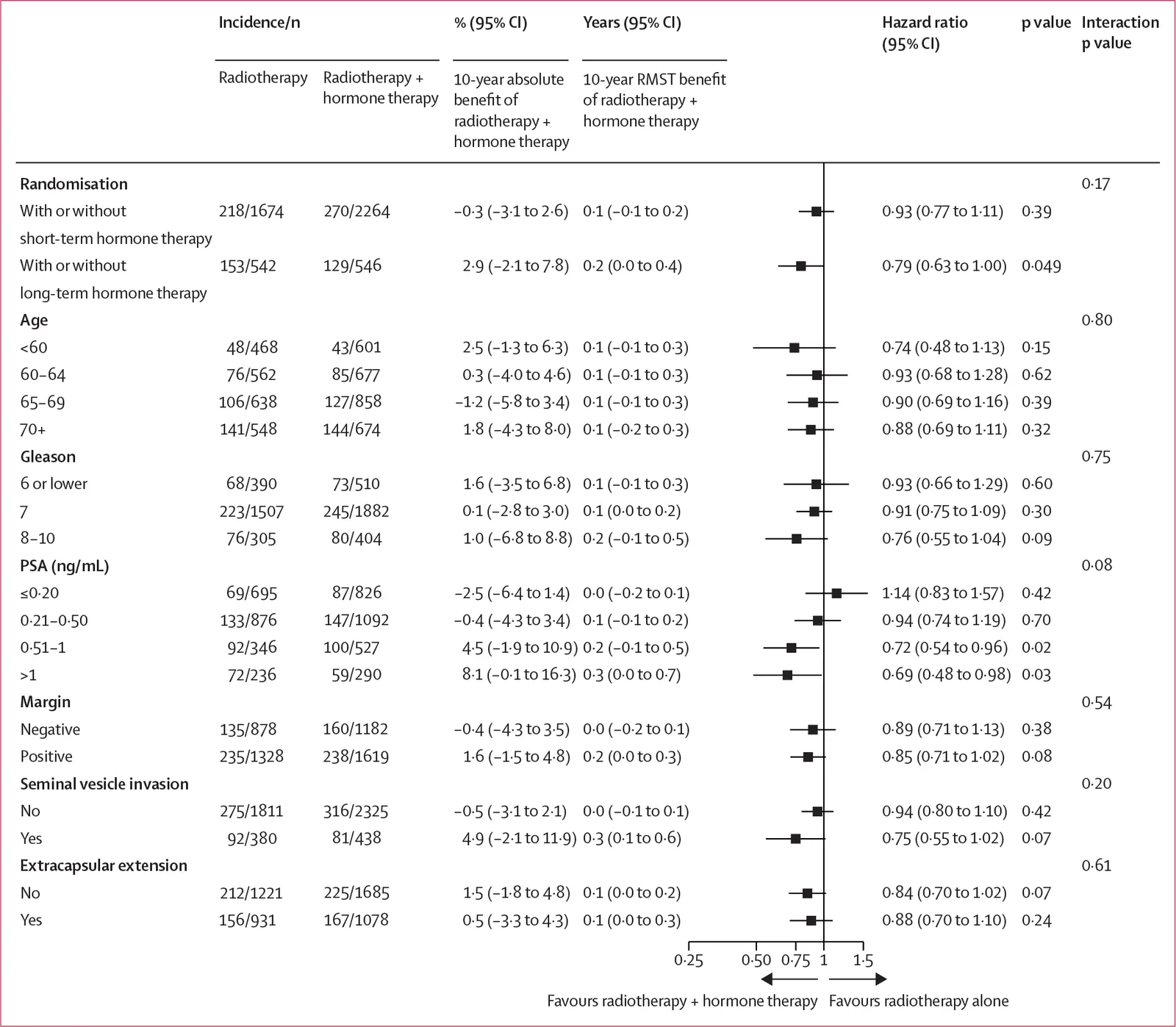
Forest plot showing effect of adding hormonal therapy to post-prostatectomy radiotherapy on overall survival, stratified by subgroups of interest n=number at risk The with or without short-term hormone therapy subgroup includes data from GETUG-AFU 16, NRG/RTOG 0534, RADICALS-HD (two-way), and RADICALS-HD (three-way). The with or without long-term hormone therapy subgroup includes data from RTOG 9601 and RADICALS-HD (three-way). For RADICALS-HD (three-way), the control group contributes to the estimation of the HR in both subgroups to represent the distinct randomisations. The interaction p value comparing treatment effects between with or without hormone therapy and with or without-hormone therapy randomisations was calculated via a split control approach^19^ to avoid unit-of-analysis error; subgroup-specific estimates use the full common control group to maximise precision for clinical interpretation. PSA=prostate-specific antigen. RMST=restricted mean survival time.

**Figure 4:**
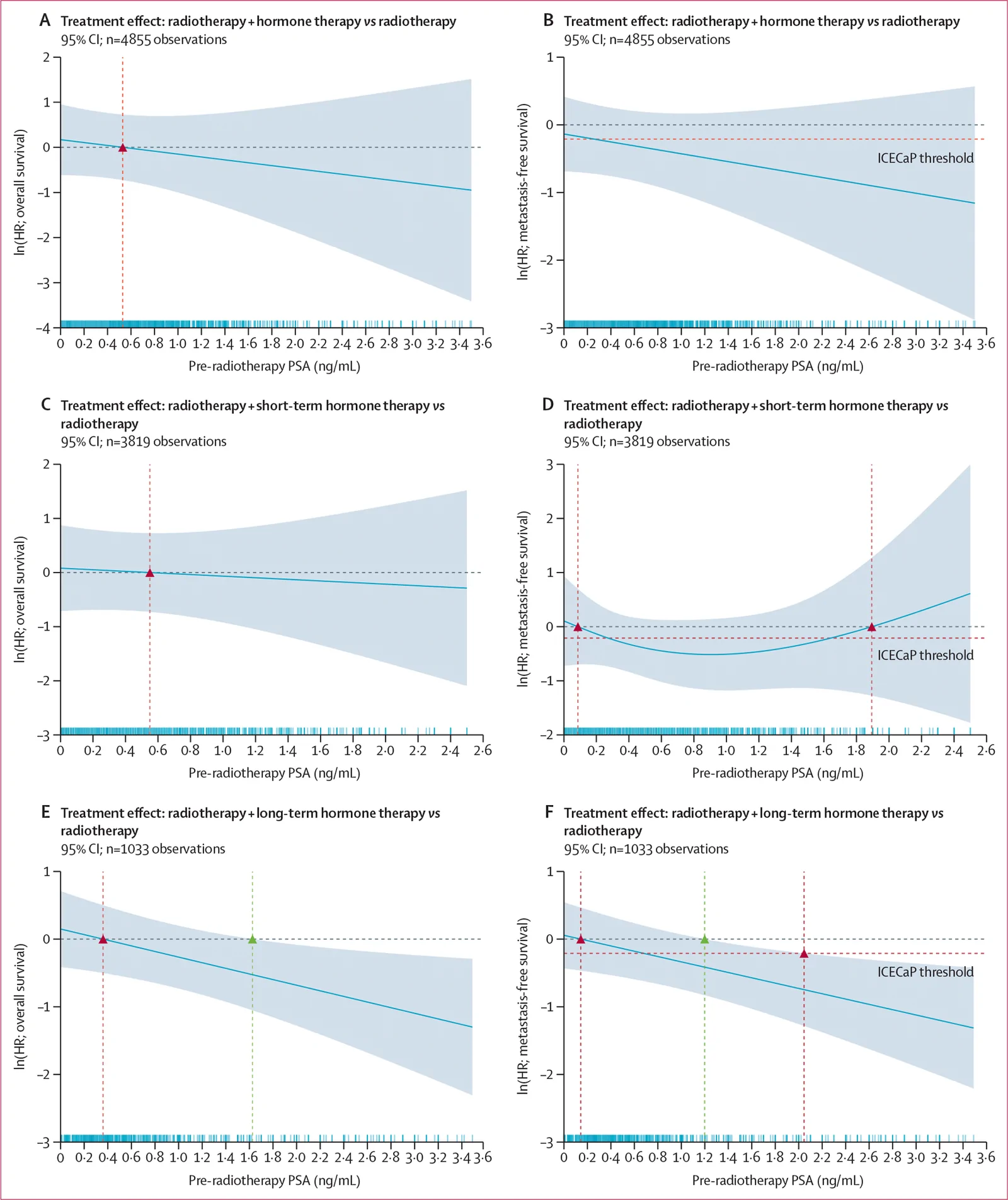
Natural cubic splines showing associations between pre-post-prostatectomy radiotherapy PSA and effect on overall survival and metastasis-free survival from adding hormonal therapy to radiotherapy Cubic splines show associations between ln(HR) and pre-post-prostatectomy radiotherapy PSA in the context of overall survival (A, C, E) and metastasis-free survival (B, D, F). In all panels, the superimposed grey dashed vertical line with a triangle indicates the pre-PORT PSA at which ln(HR)=0, or the hazard ratio=1. In panels B, D, and F, the additional superimposed red dashed vertical line with a circle indicates the pre-PORT PSA at which the upper bounds of the 95% CI for the ln(HR)=−0·21, or upper bounds for the HR=0·81. This is the ICECaP threshold for an effect size of improving metastasis-free survival that would translate to a subsequent significant improvement in overall survival. In panels E and F, an additional green triangle on the x-axis indicates the pre-PORT PSA at which the upper bound of the 95% CI for ln(HR) equals 0 (ie, upper bound of HR=1·0). PSA=prostate-specific antigen. ICECaP=Intermediate Clinical Endpoints in Cancer of the Prostate.

**Figure 5:**
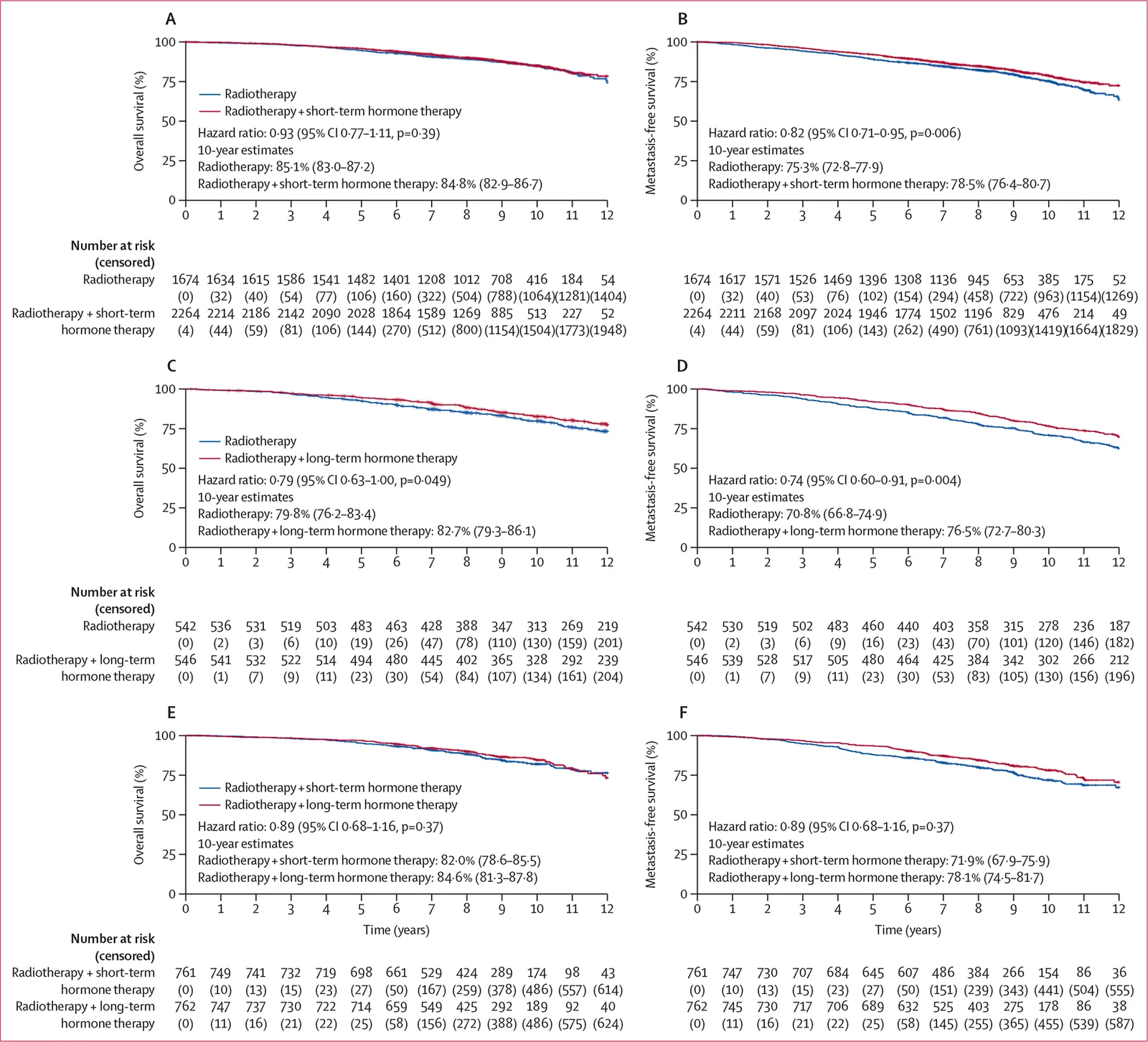
Exploratory analyses of survival effect of adding short-term and long-term hormonal therapy to post-prostatectomy radiotherapy (A, B) Kaplan–Meier survival curves showing overall survival and metastasis-free survival effect of adding short-term hormonal therapy to post-prostatectomy radiotherapy. (C, D) Kaplan–Meier survival curves showing overall survival and MFS effect of adding long-term hormonal therapy to post-prostatectomy radiotherapy. (E, F) Kaplan–Meier survival curves showing overall survival and MFS effect of prolonging short-term hormone therapy to long-term hormone therapy with post-prostatectomy radiotherapy.

**Table:** Patient characteristics

	Hormone therapy use		Short-term hormone therapy use		Long-term hormone therapy use	
	Control radiotherapy alone (n=2216)	Experimental radiotherapy + hormone therapy (n=2810)	Control radiotherapy alone (n=1674)	Experimental radiotherapy + short-term hormone therapy (n=2264)	Control radiotherapy alone (n=542)	Experimental radiotherapy + long-term hormone therapy (n=546)

Follow-up, years	9·10 (7·30–10·97)	9·10 (7·30–10·97)	8·80 (7·13–10·10)	8·80 (7·13–10·10)	12·34 (9·37–13·86)	12·34 (9·37–13·86)
Age, years						
<60	468 (21·1%)	601 (21·4%)	345 (20·6%)	478 (21·1%)	123 (22·7%)	123 (22·5%)
60–64	562 (25·4%)	677 (24·1%)	415 (24·8%)	554 (24·5%)	147 (27·1%)	123 (22·5%)
65–69	638 (28·8%)	858 (30·5%)	494 (29·5%)	691 (30·5%)	144 (26·6%)	167 (30·6%)
>70	548 (24·7%)	674 (24·0%)	420 (25·1%)	541 (23·9%)	128 (23·6%)	133 (24·4%)
Prostate-specific antigen, ng/mL	0·3 (0·2–0·6)	0·3 (0·2–0·6)	0·3 (0·2–0·5)	0·3 (0·2–0·5)	0·5 (0·3–0·9)	0·5 (0·3–0·9)
Pathological Gleason score						
6	390 (17·6%)	510 (18·1%)	270 (16·1%)	384 (17·0%)	120 (22·7%)	126 (23·7%)
7	1507 (68·0%)	1882 (67·0%)	1188 (71·0%)	1562 (69·0%)	319 (60·4%)	320 (60·2%)
8–10	305 (13·8%)	404 (14·4%)	216 (12·9%)	318 (14·0%)	89 (16·9%)	86 (16·2%)
Pathological tumour stage						
2	962 (43·4%)	1311 (46·9%)	791 (47·4%)	1122 (49·8%)	171 (31·6%)	189 (34·7%)
3a	833 (37·6%)	970 (34·6%)	623 (37·3%)	758 (33·6%)	210 (38·7%)	212 (39·0%)
3b	366 (16·5%)	439 (15·7%)	241 (14·4%)	337 (14·9%)	125 (23·1%)	102 (18·8%)
4	3 (0·1%)	9 (0·3%)	3 (0·2%)	6 (0·3%)	0	3 (0·6%)
Surgical margin status						
Negative	878 (39·8%)	1182 (42·2%)	726 (43·6%)	1030 (45·7%)	152 (28·0%)	152 (27·8%)
Positive	1328 (60·2%)	1619 (57·8%)	938 (56·4%)	1225 (54·3%)	390 (72·0%)	394 (72·2%)
Seminal vesicle invasion						
No	1811 (82·7%)	2325 (84·1%)	1406 (84·9%)	1888 (84·7%)	405 (75·7%)	437 (81·8%)
Yes	380 (17·3%)	438 (15·9%)	250 (15·1%)	341 (15·3%)	130 (24·3%)	97 (18·2%)
Extracapsular extension						
No	1221 (56·7%)	1685 (61·0%)	924 (57·0%)	1387 (62·4%)	297 (55·9%)	298 (55·3%)
Yes	931 (43·3%)	1078 (39·0%)	697 (43·0%)	837 (37·6%)	234 (44·1%)	241 (44·7%)

Data are n (%).

## Data Availability

Individual patient data obtained via data sharing agreements were used in this analysis. Researchers interested in obtaining de-identified patient data with a data dictionary from this study can contact the corresponding author via email. This might require a signed data access agreement.

## Research in context

### Evidence before this study

Prostate cancer can recur after radical prostatectomy, which can prompt postoperative radiotherapy (PORT) to the prostate bed with or without pelvic lymph nodes. Although the addition of hormonal therapy to definitive radiotherapy for intact prostate cancer has been shown to improve overall survival, the overall survival benefit of adding hormonal therapy to PORT for prostate cancer remains unclear. There is a similar lack of clarity with regards to the separate benefit of short-term (4–6 months) versus long-term (24 months) hormone therapy. We searched MEDLINE, Embase, trial registries, and major conference proceedings to retrieve randomised trials comparing PORT versus PORT plus hormone therapy with available survival data. We did a systematic literature search in the English language using MEDLINE (Jan 1, 1966, to Dec 15, 2024), Embase (Jan 1, 1982, to Dec 15, 2024), trial registries (Cochrane Central Register of Controlled Trials and ClinicalTrials.gov), the Web of Science, Scopus, and major urology and oncology conference proceedings (Jan 1, 1990, to Dec 15, 2024) using the following search terms: “randomized AND prostate” AND (“androgen deprivation” OR “hormone therapy”) AND (“radiotherapy” OR “radiation”) AND (“surgery” OR “prostatectomy”). We identified six trials with detailed overall survival and metastasis-free survival data. The six trials reached differing conclusions about the benefit of adding hormone therapy in general as well as the specific benefits of short-term and long-term hormone therapy.

### Added value of this study

This meta-analysis included individual patient data from six published trials comparing PORT to PORT plus hormone therapy and is the first study, to our knowledge, that quantitatively and comprehensively evaluates the benefit of hormone therapy, short-term, and long-term hormone therapy. It provides high-level evidence that the addition of hormone therapy to PORT does not significantly improve overall survival, with no significant interaction between the duration of hormone therapy and this effect. However, there was a significant interaction effect based on pre-PORT prostate specific antigen (PSA), with a meaningful effect potentially seen for men with pre-PORT PSAs greater than 0·5 ng/mL. An exploratory analysis of non-linear associations suggested that the addition of short-term hormone therapy to PORT would not have a significant effect on overall survival for any pre-PORT PSA, while the addition of long-term hormone therapy to PORT might significantly improve overall survival for very high pre-PORT PSA levels.

### Implications of all the available evidence

Our findings provide the strongest evidence to date suggesting that there might be no meaningful overall survival benefit to adding any form of hormone therapy, whether short-term or long-term, to PORT for men with PSA of 0·5 ng/mL or less. Furthermore, even long-term hormone therapy would only be expected to increase overall survival for men with pre-PORT PSA levels that are significantly higher than those routinely encountered in a modern PORT setting (ie, >1·6 ng/mL). Biomarkers are needed to further guide the use of hormone therapy with PORT.
