# Supplementary material for: Hormone therapy use and duration with postoperative radiotherapy for recurrent prostate cancer: an individual patient data meta-analysis
Source: Lancet. Author manuscript; Available in PMC 2026 Oct 5. (PMC13637963; doi:10.1016/S0140-6736(26)00137-6)
Supplement: Supplementary file 1 [file NIHMS2208523-supplement-Appendix__M_18480_.pdf]

THE LANCET

Supplementary appendix

This appendix formed part of the original submission and has been peer reviewed. We post it as supplied by the authors.

Supplement to: Kishan AU, Sun Y, C Parker CC, et al. Hormone therapy use and duration with postoperative radiotherapy for recurrent prostate cancer: an individual patient data meta-analysis. *Lancet* 2026; published online Feb 26. https://doi.org/10.1016/S0140-6736(26)00137-6.

Appendix

Supplementary Methods

Statistical Analysis Plan
PRISMA-IPD Checklist

Supplementary Tables

Supplementary Table 1. Trials Included in Meta-Analysis

Supplementary Table 2. Detailed Definition of Biochemical Recurrence and Distant Metastasis

Supplementary Table 3. Details on Hormonal Therapy (HT) Dosing Formulations

Supplementary Table 4. Inter-Trial Heterogeneity Assessment

Supplementary Table 5. Patient Characteristics for HT Prolongation Meta-Analysis

Supplementary Table 6. Numbers Needed to Treat for Various Hormonal Therapy (HT) Intensification Strategies

Supplementary Figures

Supplementary Figure 1. Forest plot showing trial-specific effects of hormonal therapy (HT) use on overall survival (OS), metastasis-free survival (MFS), event-free survival (EFS), and biochemical recurrence (BCR).

Supplementary Figure 2. Forest plot showing impact of adding hormonal therapy (HT) to post-prostatectomy radiotherapy (RT) on metastasis-free survival (MFS), stratified by subgroups of interest

Supplementary Figure 3. Metastasis-free survival impact of adding hormonal therapy to post-prostatectomy radiotherapy based on pre-radiotherapy PSA strata.

Supplementary Figure 4. Forest plot showing trial-specific effects of short-term hormonal therapy (ST-HT) use on overall survival (OS), metastasis-free survival (MFS), event-free survival (EFS), and biochemical recurrence (BCR).

Supplementary Figure 5. Forest plot showing impact of adding short-term hormonal therapy (ST-HT) to post-prostatectomy radiotherapy (RT) on overall survival and metastasis-free survival, stratified by subgroups of interest

Supplementary Figure 6. Forest plot showing trial-specific effects of long-term hormonal therapy (HT) use on overall survival (OS), metastasis-free survival (MFS), event-free survival (EFS), and biochemical recurrence (BCR).

Supplementary Figure 7. Forest plot showing impact of adding short-term hormonal therapy (LT-HT) to post-prostatectomy radiotherapy (RT) on overall survival and metastasis-free survival, stratified by subgroups of interest

Supplementary Figure 8. Forest plot showing trial-specific effects of long-term hormonal therapy (HT) use versus short-term hormonal therapy use on overall survival (OS), metastasis-free survival (MFS), event-free survival (EFS), and biochemical recurrence (BCR).

Supplementary Figure 9. Forest plot showing impact of prolonging short-term hormonal therapy (ST-HT) to long-term hormonal therapy (LT-HT) with post-prostatectomy radiotherapy (RT) on overall survival and metastasis-free survival, stratified by subgroups of interest.

Supplementary Methods
Statistical Analysis Plan
Study Title: POSEIDON: Post Operative Salvage or Adjuvant RadiothErapy and AntI-AnDrogen Therapy for Men with PrOstate CaNcer Meta-Analysis

Objective: The proposed individual patient data (IPD) meta-analysis aims to evaluate the benefit of adding hormonal therapy (HT) of varying durations (short-term and long-term) to postoperative radiotherapy (RT) for prostate cancer. We hypothesize that the addition of HT to postoperative RT will not improve overall survival (OS) in the overall population, but that the benefit may be modulated based on pre-PORT PSA levels.

Study Cohorts: We will utilize data from GETUG 16, NRG/RTOG 0534, and RADICALS-HD (0 vs. 6 months) and RADICALS-HD (0 vs. 6 months from 3-way) for RT alone vs. RT + short-term HT; RTOG 9601 and RADICALS-HD (0 vs. 24 months from 3-way) for RT alone vs. RT + long-term HT; and RADICALS-HD (6 vs. 24 months from 3-way) for RT + short-term HT vs. RT + long-term HT.

Data Management: The data will be pooled from the individual trials and harmonized to ensure consistency in variable definitions and analysis metrics. The individual patient data will be securely stored on servers hosted by Case Western Reserve University and the University of California, Los Angeles. Access to the data is restricted to the Principal Investigators (Drs. Dan Spratt and Amar Kishan) and the project statistician (Yilun Sun, Tahmineh Romero).

Endpoints: The primary endpoint is overall survival (OS). Secondary endpoints include metastasis-free survival (MFS), biochemical recurrence (BCR), and event-free survival (EFS). EFS is defined as the composite time to biochemical recurrence, clinical progression, initiation of salvage treatment, or death from any cause. BCR will be based on individual protocol to leverage the exact event date.

Statistical Analysis: The IPD meta-analysis will be conducted using a 1-stage approach. Stratified Cox proportional hazards models will compare the treatment groups using hazard ratios (HR) with 95% confidence intervals (CIs). Stratified Fine-Gray regression will be used to quantify treatment effects for BCR (with death as a competing risk) in terms of subdistribution hazard ratios with 95% CIs. In both models, stratified baseline hazards by trial will account for participant clustering within studies. The proportional hazards assumption will be formally tested using Grambsch-Therneau residuals.

To supplement relative risks, we will calculate the 10-year restricted mean survival time (RMST), restricted mean time lost (RMTL), and Numbers Needed to Treat (NNT). Between-trial heterogeneity will be assessed using a 2-stage random-effects meta-analysis based on the generic inverse variance method, quantifying heterogeneity using tau2 (estimated via REML), Cochran's Q (with test p-value), I2 and prediction intervals.

Subgroup Analysis: We will evaluate treatment effects in the following predefined subgroups:

1. **Pre-radiotherapy PSA:** Analyzed as (a) Continuous variable (subject to outliers); (b) Binary (≤0.5 vs. >0.5 ng/mL, primary analysis); and (c) Categorical (≤0.20 vs. 0.21-0.5 vs. 0.51-1.0 vs. >1.0 ng/mL, secondary, in case some bins will have low counts) to distinguish very early salvage.
2. **Pathologic Gleason score:** (6 vs. 7 vs. 8-10).
3. **Age:** (<60 vs. 60-64 vs. 65-69 vs. >70).
4. **Margin status:** (Positive vs. Negative).
5. **Seminal vesicle invasion:** (Present vs. Absent).
6. **Extracapsular extension:** (Present vs. Absent).

Note if the obtained IPD already and only have these variables categorized in other ways, the final cut-off might differ aiming to leverage as much data as possible.

We will also assess the effect difference between randomization schemes: +/- LTHT vs. +/-STHT. For this analysis only, the RADICALS-HD (3-way) trial control arm will contribute to the estimation of the hazard ratio in both subgroups. These will be treated as separate strata to represent the distinct randomizations, ensuring accurate estimation of the interaction term without double-counting patients in the overall efficacy analysis.

Interaction terms will be included in the stratified regression models to assess the differential effects of HT across subgroups. One covariate/subgroup will be assessed each time, with ~ Trt + Covariate + Trt* Covariate. We will fit the models with stratified baseline hazard, fixed effects include stratified treatment and covariates' main effects, common interaction effect; random effect includes the interaction random slope only. This specification follows the approach ii in section 7.4.2.2. in Riley et al. (2021). Given the limited number of trials k<10, our previous MARCAP experience suggest this approach (or alternative approach i from the same reference) is likely unstable; therefore, standard one-stage stratified models are pre-specified in case the model convergence failed. Without specification otherwise, a global test will be used for categorical/ordinal variables without assuming a linear trend.

Exploratory Analysis: Non-linear associations between pre-RT PSA and outcomes will be modeled using natural cubic splines, with the number of knots selected based on the lowest Akaike Information Criterion (AIC). In addition, a comparison of ST-HT versus LT-HT will be performed restricted to trials directly randomizing these durations (RADICALS-HD) as part of explorations. Additionally, we will assess the surrogate threshold effect for MFS on OS using the established ICECAP threshold (HR < 0.81).

Missing Data: We anticipate minimal missing data (<5%) for baseline covariates. If confirmed, and if missingness is balanced between arms, a complete-case analysis will be performed.

Software: All analyses will be conducted on an intention-to-treat basis using R (version 4.3.2) with a two-tailed significance level of 0.05. Due to the exploratory nature of the subgroup analyses and high correlation between endpoints, no formal adjustment for multiple testing will be performed.

Reference

Riley RD, Stewart LA, Tierney JF. Individual participant data meta-analysis for healthcare research. Individual Participant Data Meta-Analysis: a handbook for healthcare research. 2021 Apr 22:1-6.

PRISMA IPD Checklist

PRISMA-IPD Checklist of items to include when reporting a systematic review and meta-analysis of individual participant data (IPD)

PRISMA-IPD Section/topic	Item No	Checklist item	Reported on page
**Title**			
Title	1	Identify the report as a systematic review and meta-analysis of individual participant data.	1
**Abstract**			
Structured summary	2	Provide a structured summary including as applicable:	2
		**Background**: state research question and main objectives, with information on participants, interventions, comparators and outcomes.	
		**Methods**: report eligibility criteria; data sources including dates of last bibliographic search or elicitation, noting that IPD were sought; methods of assessing risk of bias.	
		**Results**: provide number and type of studies and participants identified and number (%) obtained; summary effect estimates for main outcomes (benefits and harms) with confidence intervals and measures of statistical heterogeneity. Describe the direction and size of summary effects in terms meaningful to those who would put findings into practice.	
		**Discussion:** state main strengths and limitations of the evidence, general interpretation of the results and any important implications.	
		**Other:** report primary funding source, registration number and registry name for the systematic review and IPD meta-analysis.	
**Introduction**			
Rationale	3	Describe the rationale for the review in the context of what is already known.	4
Objectives	4	Provide an explicit statement of the questions being addressed with reference, as applicable, to participants, interventions, comparisons, outcomes and study design (PICOS). Include any hypotheses that relate to particular types of participant-level subgroups.	4-5
**Methods**			
Protocol and registration	5	Indicate if a protocol exists and where it can be accessed. If available, provide registration information including registration number and registry name. Provide publication details, if applicable.	9
Eligibility criteria	6	Specify inclusion and exclusion criteria including those relating to participants, interventions, comparisons, outcomes, study design and characteristics (e.g. years when conducted, required minimum follow-up). Note whether these were applied at the study or individual level i.e. whether eligible participants were included (and ineligible participants excluded) from a study that included a wider population than specified by the review inclusion criteria. The rationale for criteria should be stated.	6
Identifying studies - information sources	7	Describe all methods of identifying published and unpublished studies including, as applicable: which bibliographic databases were searched with dates of coverage; details of any hand searching including of conference proceedings; use of study registers and agency or company databases;	6

		contact with the original research team and experts in the field; open adverts and surveys. Give the date of last search or elicitation.	
Identifying studies - search	8	Present the full electronic search strategy for at least one database, including any limits used, such that it could be repeated.	6
Study selection processes	9	State the process for determining which studies were eligible for inclusion.	6
Data collection processes	10	Describe how IPD were requested, collected and managed, including any processes for querying and confirming data with investigators. If IPD were not sought from any eligible study, the reason for this should be stated (for each such study).	6
		If applicable, describe how any studies for which IPD were not available were dealt with. This should include whether, how and what aggregate data were sought or extracted from study reports and publications (such as extracting data independently in duplicate) and any processes for obtaining and confirming these data with investigators.	
Data items	11	Describe how the information and variables to be collected were chosen. List and define all study level and participant level data that were sought, including baseline and follow-up information. If applicable, describe methods of standardising or translating variables within the IPD datasets to ensure common scales or measurements across studies.	7
IPD integrity	A1	Describe what aspects of IPD were subject to data checking (such as sequence generation, data consistency and completeness, baseline imbalance) and how this was done.	7
Risk of bias assessment in individual studies.	12	Describe methods used to assess risk of bias in the individual studies and whether this was applied separately for each outcome. If applicable, describe how findings of IPD checking were used to inform the assessment. Report if and how risk of bias assessment was used in any data synthesis.	7
Specification of outcomes and effect measures	13	State all treatment comparisons of interests. State all outcomes addressed and define them in detail. State whether they were pre-specified for the review and, if applicable, whether they were primary/main or secondary/additional outcomes. Give the principal measures of effect (such as risk ratio, hazard ratio, difference in means) used for each outcome.	7-8
Synthesis methods	14	Describe the meta-analysis methods used to synthesise IPD. Specify any statistical methods and models used. Issues should include (but are not restricted to):<br>• Use of a one-stage or two-stage approach.<br>• How effect estimates were generated separately within each study and combined across studies (where applicable).<br>• Specification of one-stage models (where applicable) including how clustering of patients within studies was accounted for.<br>• Use of fixed or random effects models and any other model assumptions, such as proportional hazards.<br>• How (summary) survival curves were generated (where applicable).<br>• Methods for quantifying statistical heterogeneity (such as I^2 and τ^2).<br>• How studies providing IPD and not providing IPD were analysed together (where applicable).	8-12

		• How missing data within the IPD were dealt with (where applicable).	
Exploration of variation in effects	A2	If applicable, describe any methods used to explore variation in effects by study or participant level characteristics (such as estimation of interactions between effect and covariates). State all participant-level characteristics that were analysed as potential effect modifiers, and whether these were pre-specified.	8
Risk of bias across studies	15	Specify any assessment of risk of bias relating to the accumulated body of evidence, including any pertaining to not obtaining IPD for particular studies, outcomes or other variables.	11
Additional analyses	16	Describe methods of any additional analyses, including sensitivity analyses. State which of these were pre-specified.	8-12
**Results**			
Study selection and IPD obtained	17	Give numbers of studies screened, assessed for eligibility, and included in the systematic review with reasons for exclusions at each stage. Indicate the number of studies and participants for which IPD were sought and for which IPD were obtained. For those studies where IPD were not available, give the numbers of studies and participants for which aggregate data were available. Report reasons for non-availability of IPD. Include a flow diagram.	13
Study characteristics	18	For each study, present information on key study and participant characteristics (such as description of interventions, numbers of participants, demographic data, unavailability of outcomes, funding source, and if applicable duration of follow-up). Provide (main) citations for each study. Where applicable, also report similar study characteristics for any studies not providing IPD.	13
IPD integrity	A3	Report any important issues identified in checking IPD or state that there were none.	13
Risk of bias within studies	19	Present data on risk of bias assessments. If applicable, describe whether data checking led to the up-weighting or down-weighting of these assessments. Consider how any potential bias impacts on the robustness of meta-analysis conclusions.	13
Results of individual studies	20	For each comparison and for each main outcome (benefit or harm), for each individual study report the number of eligible participants for which data were obtained and show simple summary data for each intervention group (including, where applicable, the number of events), effect estimates and confidence intervals. These may be tabulated or included on a forest plot.	Supplement
Results of syntheses	21	Present summary effects for each meta-analysis undertaken, including confidence intervals and measures of statistical heterogeneity. State whether the analysis was pre-specified, and report the numbers of studies and participants and, where applicable, the number of events on which it is based.	13-16
		When exploring variation in effects due to patient or study characteristics, present summary interaction estimates for each characteristic examined, including confidence intervals and measures of statistical heterogeneity. State whether the analysis was pre-specified. State whether any interaction is consistent across trials.	
		Provide a description of the direction and size of effect in terms meaningful to those who would put findings into practice.	

Risk of bias across studies	22	Present results of any assessment of risk of bias relating to the accumulated body of evidence, including any pertaining to the availability and representativeness of available studies, outcomes or other variables.	13
Additional analyses	23	Give results of any additional analyses (e.g. sensitivity analyses). If applicable, this should also include any analyses that incorporate aggregate data for studies that do not have IPD. If applicable, summarise the main meta-analysis results following the inclusion or exclusion of studies for which IPD were not available.	13-16
**Discussion**			
Summary of evidence	24	Summarise the main findings, including the strength of evidence for each main outcome.	17
Strengths and limitations	25	Discuss any important strengths and limitations of the evidence including the benefits of access to IPD and any limitations arising from IPD that were not available.	20-21
Conclusions	26	Provide a general interpretation of the findings in the context of other evidence.	21-22
Implications	A4	Consider relevance to key groups (such as policy makers, service providers and service users). Consider implications for future research.	22
**Funding**			
Funding	27	Describe sources of funding and other support (such as supply of IPD), and the role in the systematic review of those providing such support.	25

A1 – A3 denote new items that are additional to standard PRISMA items. A4 has been created as a result of re-arranging content of the standard PRISMA statement to suit the way that systematic review IPD meta-analyses are reported.

© Reproduced with permission of the PRISMA IPD Group, which encourages sharing and reuse for non-commercial purposes

Supplementary Tables

Supplementary Table 1. Trials Included in Meta-Analysis

	Population	Dates	Experimental Arm	Control Arm	RT Dose*	Participants	Controls	OS Result*
**RT+ST-HT**								
GETUG-16[1]	pT2-pT4a, PSA 0.2-2.0 ng/mL	2006-2010	RT+6 months HT	RT	66 Gy	369	374	0.93 (0.63-1.39)
RADICALS (short versus none)[2]	Due to receive post-operative RT	2007-2015	RT+6 months HT	RT	66 Gy or 52.5 Gy	743	747	0.88 (0.65-1.19)
RADICALS (3-way)[3]	Due to receive post-operative RT	2007-2015	RT+6 months HT	RT	66 Gy or 52.5 Gy	164	166	1.12 (0.59-2.16)
RTOG 0534[4]	pT2-T3, GS 9 or less, PSA 0.1-2.0 ng/mL	2008-2015	RT+4-6 months HT	RT alone	64.8–70.2 Gy	1200	592	0.89 (0.60-1.31)** 0.94 (0.64-1.37)
**RT+LT-HT**								
RTOG 9601[5]	pT2-T3, PSA 0.2-4.0 ng/mL	1998-2003	RT+24 months HT	RT alone	64.8 Gy	384	376	0.77 (0.59-0.99)
RADICALS (3-Way)[3]	Due to receive post-operative RT	2007-2015	RT+24 months HT	RT alone	66 Gy or 52.5 Gy	162	166	1.07 (0.53-2.12)
**RT+ST-HT versus RT+LT-HT**								
RADICALS (long versus short)[6]	Due to receive post-operative RT	2007-2015	RT+24 months HT	RT+6 months HT	66 Gy or 52.5 Gy	762	761	0.88 (0.66-1.17)
RADICALS (3-way)[3]	Due to receive post-operative RT	2007-2015	RT+24 months HT	RT+6 months HT	66 Gy or 52.5 Gy	162	164	***

*Results shown as hazard ratio (95% confidence interval), calculated directly from the harmonized individual patient data (IPD).
**In RTOG 0534, two arms included ST-HT, with one also exploring the addition of pelvic radiotherapy. For this meta-analysis, both arms with ST-HT have been collapsed into a single experimental arm. The two provided OS hazard ratios correspond to the HR for salvage radiotherapy to the bed alone + ST-HT versus salvage radiotherapy to the bed alone, and the second is the HR for salvage radiotherapy to the bed and nodes + ST-HT versus salvage radiotherapy to the bed alone.

***The original publication of the 3-way comparison did not report an OS hazard ratio.
Abbreviations: GETUG, Groupe d'Etude des Tumeurs Uro-Genitales; GS, Gleason score; Gy, gray; HT, hormonal therapy; PSA, prostate-specific antigen; pT, pathologic tumor stage; OS, overall survival; RADICALS, Radiotherapy and Androgen Deprivation In Combination After Local Surgery; RT, radiotherapy; RTOG, Radiation Therapy Oncology Group

Supplementary Table 2. Detailed Definition of Biochemical Recurrence and Distant Metastasis

Trial	Biochemical Recurrence	Distant Metastasis
GETUG-AFU-16[1]	An increase in PSA concentration by more than 0·5 ng/mL compared with the nadir (confirmed by a second PSA measurement)	A metastatic event was defined as a progression outside of the prostate bed, therefore any nodal relapse was considered to be a metastatic relapse.
RADICALS (short versus none)[2]	PSA of 0·4 ng/mL or greater following postoperative radiotherapy, or PSA of more than 2·0 ng/mL at any time	Distant metastases could be bone, liver, lung, distant node, or other metastases, but did not include pelvic nodes.
RADICALS (long versus short)[6]	PSA of 0·4 ng/mL or greater following postoperative radiotherapy, or PSA of more than 2·0 ng/mL at any time	Distant metastases could be bone, liver, lung, distant node, or other metastases, but did not include pelvic nodes.
RADICALS (3-way)[3]	PSA of 0·4 ng/mL or greater following postoperative radiotherapy, or PSA of more than 2·0 ng/mL at any time	Distant metastases could be bone, liver, lung, distant node, or other metastases, but did not include pelvic nodes.
NRG/RTOG 0534[4]	PSA ≥2 ng/mL over the nadir PSA	Distant metastases could be bone, liver, lung, distant node, or other metastases, but did not include pelvic nodes.
NRG/RTOG 9601[5]	An increase of at least 0.3 ng per milliliter above the lowest detectable PSA level after protocol treatment or as the initiation of any subsequent hormone therapy	Distant metastases could be bone, liver, lung, distant node, or other metastases, but did not include pelvic nodes.

Abbreviations: GETUG, Groupe d'Etude des Tumeurs Uro-Genitales; RADICALS, Radiotherapy and Androgen Deprivation In Combination After Local Surgery; RTOG, Radiation Therapy Oncology Group

Supplementary Table 3. Details on Hormonal Therapy (HT) Dosing Formulations

Trial	Details on Hormonal Therapy
GETUG-AFU-16[1]	6 months of goserelin, given q3 monthly beginning the first day of RT
RADICALS (short versus none)[2]	6 months of GnRH agonist (84%) or bicalutamide 150 mg (16%); GnRH preferably dosed monthly and given with one month of anti-androgen. HT begins 2 months before RT
RADICALS (long versus short)[6]	RT+ Short-Term HT Arm: 6 months of GnRH agonist (84%) or bicalutamide 150 mg (16%); GnRH preferably dosed monthly and given with one month of anti-androgen. HT begins 2 months before RT<br><br>RT+ Long-term HT Arm: 24 months of GnRH agonist (84%) or bicalutamide (16%); GnRH preferably dosed q3monthly and given with one month of anti-androgen. HT begins 2 months before RT
RADICALS (3-way)[3]	RT+ Short-Term HT Arm: 6 months of GnRH agonist (87%) or bicalutamide 150 mg (13%); GnRH preferably dosed monthly and given with one month of anti-androgen. HT begins 2 months before RT<br><br>RT+ Long-term HT Arm: 24 months of GnRH agonist (86%) or bicalutamide (14%); GnRH preferably dosed q3monthly and given with one month of anti-androgen. HT begins 2 months before RT
NRG/RTOG 0534[4]	4-6 months of GnRH agonist with 4 months of nonsteroidal antiandrogen, beginning 2 months before RT.<br>32% received 4 months of HT, 2% received 5 months, and 60% received 6 months; remainder received less (2%) or more (4%) than protocol duration
NRG/RTOG 9601[5]	24 months of 150 mg bicalutamide daily, beginning at the start of RT

Abbreviations: GETUG, Groupe d'Etude des Tumeurs Uro-Genitales; GnRH, gonadotropin-releasing hormone; HT, hormonal therapy; RADICALS, Radiotherapy and Androgen Deprivation In Combination After Local Surgery; RT, radiation therapy; RTOG, Radiation Therapy Oncology Group

Supplementary Table 4. Inter-Trial Heterogeneity Assessment

Endpoint	Number of Trials	tau^2	I^2	Cochran's Q	p value (test for homogeneity based on Q)	Prediction Interval
**RT+HT vs RT**						
MFS	5	0	0	1.40	0.84	(0.67, 0.94)
Death	5	0	0	1.62	0.8	(0.71, 1.07)
**RT + Short-term HT vs. RT**						
MFS	4	0	0	0.99	0.8	(0.65, 1.04)
Death	4	0	0	0.20	0.98	(0.69, 1.24)
**RT + Long-term HT vs. RT**						
MFS	2	0	0	0.10	0.75	(0.19, 2.83)
Death	2	0	0	0.39	0.53	(0.17, 3.62)
**RT + Long-term HT vs. RT+ Short-term HT**						
MFS	2	0	0	0.10	0.76	(0.18, 3.23)
OS	2	0	0	0.0002	0.99	(0.15, 5.11)

HT, hormonal therapy; MFS, metastasis-free survival; RT, radiation therapy

Supplementary Table 5. Patient Characteristics for HT Prolongation Meta-Analysis

	HT Prolongation	
	**Control** RT+ST-HT	**Experimental** RT+LT-HT
**N=**	761	762
**Follow-up** (median, IQR), years	8.89 (7.03, 10.05)	
**Age**		
**<60**	171.0 (22.5%)	155.0 (20.3%)
**60-64**	182.0 (23.9%)	188.0 (24.7%)
**65-69**	262.0 (34.4%)	248.0 (32.5%)
**>70**	146.0 (19.2%)	171.0 (22.4%)
**PSA** (median, IQR) ng/mL	0.2 (0.1, 0.5)	0.2 (0.1, 0.5
**Pathologic Gleason Score**		
6	61.0 (8.0%)	53.0 (7.0%)
7	484.0 (63.7%)	490.0 (64.3%)
8-10	215.0 (28.3%)	219.0 (28.7%)
**Pathologic Tumor Stage**		
2	206.0 (27.1%)	215.0 (28.4%)
3a	327.0 (43.1%)	309.0 (40.7%)
3b	215.0 (28.3%)	220.0 (29.0%)
4	11.0 (1.4%)	15.0 (2.0%)
**Surgical Margin Status**		
Negative	281.0 (36.9%)	278.0 (36.5%)
Positive	480.0 (63.1%)	484.0 (63.5%
**Seminal Vesicle Invasion**		

No	509.0 (68.9%)	516.0 (70.1%)
Yes	230.0 (31.1%)	220.0 (29.9%)
**Extracapsular Extension**		
No	229.0 (31.5%)	237.0 (32.3%)
Yes	497.0 (68.5%)	497.0 (67.7%)

Supplementary Table 6. Numbers Needed to Treat for Various Hormonal Therapy (HT) Intensification Strategies at 10 Years

	PSA Stratum (ng/mL)			
	≤0.2	0.21-0.5	0.51-1.0	>1.0
RT + HT *versus* RT alone				
Death or Metastasis	-40 (-14, 45)*	21 (11, 913)	14 (7, 496)	7 (4, 23)
Death	-40 (-16, 72)	-224 (-23, 29)	22 (-52, 9)	12 (-800, 6)
RT+Short-term HT *versus* RT alone				
Death or Metastasis	-39 (-13, 39)	24 (-92, 10)	9 (5, 79)	7 (4, 58)
Death	-52 (-16, 44)	-56 (-16, 38)	18 (-30, 7)	12 (-30, 5)
RT+Long-term HT *versus* RT alone				
Death or Metastasis	-52 (-7, 10)	20 (-25, 7)	18 (-18, 6)	7 (4, 105)
Death	-15 (-6, 22)	45 (-17, 10)	16 (-27, 6)	13 (-23, 5)
RT+Long-term HT *versus* RT+short-term HT				
Death or Metastasis	28 (-27, 9)	16 (-20, 6)	16 (-10, 5)	7 (-46, 3)
Death	125 (-16, 13)	19 (-24, 7)	-149 (-7, 8)	25 (-11, 6)

*Note: a number needed to treat <0 indicates a "number needed to harm"

Supplementary Table 7. Sensitivity Analysis Comparing One-Stage and Two-Stage Interaction Estimates for Treatment Effect Modification

**Panel A. Addition of Hormonal Therapy to PORT (Figure 3, Supplementary Figure 2)**				
**Subgroup**	**OS (1-stage)**	**OS (2-stage)**	**MFS (1-stage)**	**MFS (2-stage)**
Age	0.80	0.45	0.92	0.81
Pathologic Gleason Score	0.75	0.65	0.41	0.25
Pre-PORT PSA	0.08	0.14	0.10	0.07
Pre-PORT PSA (>0.50 vs. ≤0.50)	0.02	0.03	0.06	0.11
Surgical Margin Status	0.54	0.81	0.48	0.43
Seminal Vesicle Invasion	0.20	0.24	0.03	0.03
Extracapsular Extension	0.61	0.80	0.34	0.63

**Panel B. Addition of Short-Term HT to PORT (Supplementary Figure 5)**				
**Subgroup**	**OS (1-stage)**	**OS (2-stage)**	**MFS (1-stage)**	**MFS (2-stage)**
Age	0.54	0.84	0.37	0.86
Pathologic Gleason Score	0.65	0.49	0.71	0.53
Pre-PORT PSA	0.56	0.50	0.29	0.10
Pre-PORT PSA (>0.50 vs. ≤0.50)	0.22	0.21	0.30	0.35
Surgical Margin Status	0.91	0.76	0.61	0.44
Seminal Vesicle Invasion	0.38	0.30	0.24	0.17
Extracapsular Extension	0.06	0.06	0.13	0.25

**Panel C. Addition of Long-Term HT to PORT (Supplementary Figure 7)**				
**Subgroup**	**OS (1-stage)**	**OS (2-stage)**	**MFS (1-stage)**	**MFS (2-stage)**
Age	0.44	0.32	0.25	0.86
Pathologic Gleason Score	0.37	0.16	0.37	0.32
Pre-PORT PSA	0.12	0.046	0.38	0.40
Pre-PORT PSA (>0.50 vs. ≤0.50)	0.049	0.06	0.14	0.16
Surgical Margin Status	0.40	0.40	0.78	0.78
Seminal Vesicle Invasion	0.52	0.54	0.05	0.06
Extracapsular Extension	0.06	0.047	0.46	0.42

**Panel D. Prolongation from Short-Term to Long-Term HT (Supplementary Figure 9)**				
**Subgroup**	**OS (1-stage)**	**OS (2-stage)**	**MFS (1-stage)**	**MFS (2-stage)**
Age	0.006	0.56	0.04	0.37
Pathologic Gleason Score	0.07	0.053	0.03	0.03

Pre-PORT PSA	0.92	0.52	0.93	0.91
Pre-PORT PSA (>0.50 vs. ≤0.50)	0.88	0.88	0.84	0.85
Surgical Margin Status	0.94	0.95	0.84	0.76
Seminal Vesicle Invasion	0.02	0.03	0.13	0.16
Extracapsular Extension	0.25	0.31	0.11	0.14
Pre-RP PSA	0.55	0.58	0.55	0.53

Abbreviations: HT, hormonal therapy; OS, overall survival; MFS, metastasis-free survival; PORT, post-operative radiotherapy; PSA, prostate-specific antigen; RP, radical prostatectomy.

Notes: P-values shown are for treatment-by-covariate interaction terms. The one-stage approach estimates interaction effects from a pooled Cox model stratified by trial. The two-stage approach first estimates within-trial interaction effects separately for each trial, then pools these estimates using random-effects meta-analysis (REML for τ^2). This sensitivity analysis was performed to assess potential aggregation bias. Pre-PORT PSA was analyzed as a categorical variable (≤0.20 vs. 0.21-0.5 vs. 0.51-1.0 vs. >1.0 ng/mL). Concordance between one-stage and two-stage estimates suggests that aggregation bias is not substantially influencing the findings.

Supplementary Figure 1. Forest plot showing trial-specific effects of hormonal therapy (HT) use on overall survival (OS), metastasis-free survival (MFS), event-free survival (EFS), and biochemical recurrence (BCR). 10-year absolute benefits as well as 10-year restricted mean survival time (RMFST) benefits are shown, along with hazard ratios (HR). 95% confidence intervals (CI 95%) are shown for all estimates, as are p-values for HRs. Outcome incidence is also shown. GETUG, Groupe d'Etude des Tumeurs Uro-Genitales; N, number of at-risk patients; RADICALS, Radiotherapy and Androgen Deprivation In Combination After Local Surgery; RTOG, Radiation Therapy Oncology Group

Endpoint	Study	RT Incidence/N	RT+HT Incidence/N	10−yr Absolute Benefit of RT+HT (%, CI 95%)	10−yr RMST Benefit of RT+HT (Years, CI 95%)	HR (95%)	p value
OS	GETUG−16	51/373	46/369	0.5 (−5.4, 6.4)	0.1 (−0.1, 0.3)	0.94 (0.63, 1.40)	0.75
	RADICALS (2−way)	76/571	69/579	0.4 (−4.6, 5.4)	0.0 (−0.2, 0.2)	0.90 (0.65, 1.25)	0.53
	RADICALS (3−way)	44/332	44/326	−1.6 (−8.4, 5.3)	0.1 (−0.2, 0.3)	0.99 (0.65, 1.51)	0.98
	RTOG−0534	69/564	132/1152	−0.2 (−5.1, 4.7)	0.1 (−0.1, 0.3)	0.91 (0.68, 1.22)	0.55
	RTOG−9601	131/376	108/384	4.0 (−1.9, 9.8)	0.3 (0.0, 0.5)	0.77 (0.59, 0.99)	0.04
	Combined	371/2216	399/2810	0.8 (−1.7, 3.2)	0.1 (0.0, 0.2)	0.87 (0.76, 1.01)	0.06
MFS	GETUG−16	106/373	77/369	6.6 (−1.0, 14.3)	0.4 (0.1, 0.7)	0.74 (0.55, 0.99)	0.04
	RADICALS (2−way)	110/571	95/579	2.3 (−3.2, 7.9)	0.1 (−0.1, 0.4)	0.85 (0.65, 1.12)	0.26
	RADICALS (3−way)	64/332	57/326	−1.3 (−8.9, 6.2)	0.0 (−0.3, 0.4)	0.88 (0.62, 1.26)	0.49
	RTOG−0534	109/564	189/1152	2.8 (−2.7, 8.3)	0.3 (0.0, 0.5)	0.82 (0.65, 1.03)	0.09
	RTOG−9601	170/376	137/384	7.3 (0.7, 13.9)	0.5 (0.2, 0.9)	0.73 (0.58, 0.92)	0.006
	Combined	559/2216	555/2810	3.8 (1.0, 6.6)	0.3 (0.1, 0.4)	0.79 (0.70, 0.89)	< 0.001
EFS	GETUG−16	187/373	120/369	14.9 (7.2, 22.7)	1.4 (0.9, 1.9)	0.56 (0.44, 0.70)	< 0.001
	RADICALS (2−way)	265/571	190/579	14.9 (8.7, 21.1)	1.1 (0.7, 1.5)	0.62 (0.52, 0.75)	< 0.001
	RADICALS (3−way)	156/332	106/326	12.9 (4.3, 21.6)	1.3 (0.8, 1.8)	0.59 (0.46, 0.75)	< 0.001
	RTOG−0534	278/564	379/1152	16.5 (10.5, 22.5)	1.4 (1.0, 1.7)	0.56 (0.48, 0.65)	< 0.001
	RTOG−9601	288/376	237/384	16.8 (9.9, 23.8)	1.6 (1.2, 2.1)	0.60 (0.50, 0.71)	< 0.001
	Combined	1174/2216	1032/2810	15.9 (12.8, 19.0)	1.4 (1.2, 1.6)	0.58 (0.54, 0.63)	< 0.001
BCR	GETUG−16	124/373	65/369	15.3 (9.0, 21.6)	1.3 (0.8, 1.8)	0.47 (0.35, 0.64)	< 0.001
	RADICALS (2−way)	221/571	142/579	14.9 (9.3, 20.5)	1.2 (0.8, 1.6)	0.56 (0.46, 0.69)	< 0.001
	RADICALS (3−way)	130/332	75/326	17.3 (9.8, 24.9)	1.4 (0.9, 1.9)	0.51 (0.38, 0.67)	< 0.001
	RTOG−0534	245/564	301/1152	18.5 (12.7, 24.4)	1.4 (1.0, 1.7)	0.51 (0.43, 0.60)	< 0.001
	RTOG−9601	252/376	169/384	24.3 (17.2, 31.3)	2.0 (1.5, 2.5)	0.48 (0.40, 0.58)	< 0.001
	Combined	972/2216	752/2810	17.7 (14.8, 20.6)	1.4 (1.2, 1.6)	0.51 (0.46, 0.56)	< 0.001

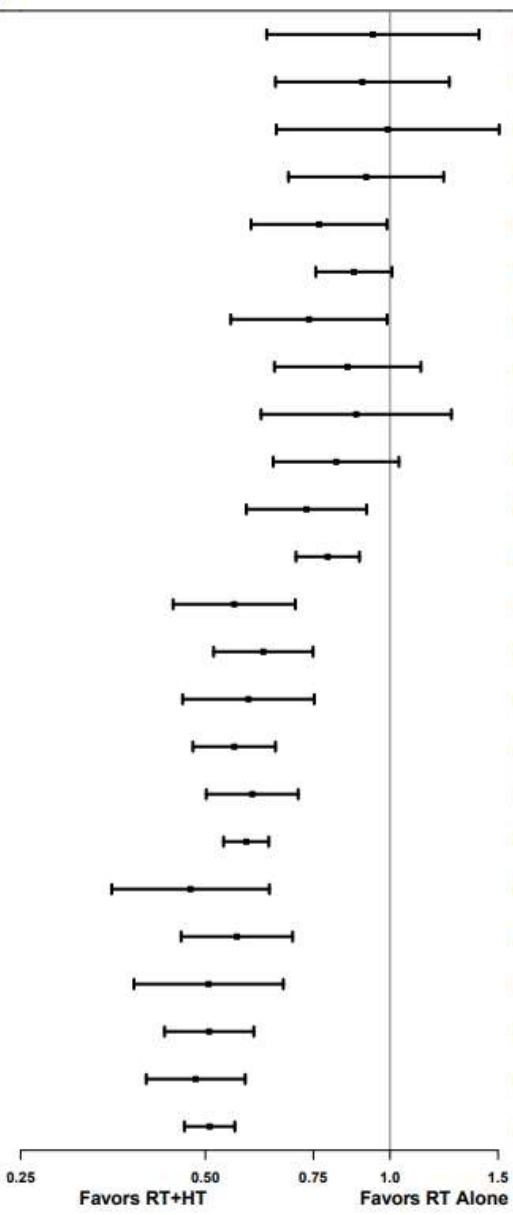

Supplementary Figure 2. Forest plot showing impact of adding hormonal therapy (HT) to post-prostatectomy radiotherapy (RT) on metastasis-free survival (MFS), stratified by subgroups of interest.

Endpoint	Age	RT Incidence/N	RT+HT Incidence/N	10−yr Absolute Benefit of RT+HT (%, CI 95%)	10−yr RMST Benefit of RT+HT (Years, CI 95%)	HR (95%)	p value	Interaction p value
Age	<60	93/468	85/601	5.4 (−0.1, 10.9)	0.3 (0.0, 0.5)	0.76 (0.56, 1.02)	0.07	0.92
	60−64	129/562	130/677	2.7 (−2.5, 8.0)	0.2 (−0.1, 0.4)	0.82 (0.64, 1.04)	0.11	
	65−69	154/638	166/858	0.6 (−4.5, 5.8)	0.3 (0.0, 0.5)	0.80 (0.64, 1.00)	0.047	
	70+	183/548	174/674	6.8 (0.2, 13.4)	0.3 (0.0, 0.6)	0.80 (0.64, 0.98)	0.04	
Gleason	6 or lower	79/390	89/510	2.6 (−3.2, 8.4)	0.1 (−0.1, 0.4)	0.91 (0.67, 1.24)	0.58	0.41
	7	356/1507	348/1882	3.7 (0.3, 7.2)	0.2 (0.1, 0.4)	0.81 (0.69, 0.94)	0.004	
	8−10	119/305	115/404	5.4 (−3.3, 14.0)	0.6 (0.2, 1.1)	0.67 (0.52, 0.87)	0.002	
PSA (ng/ml)	<= 0.20	109/695	124/826	−2.5 (−7.3, 2.2)	0.0 (−0.2, 0.2)	1.04 (0.81, 1.36)	0.76	0.10
	0.21−0.50	212/876	198/1092	4.7 (0.1, 9.3)	0.3 (0.1, 0.5)	0.78 (0.64, 0.95)	0.01	
	0.51−1	129/346	145/527	7.3 (0.2, 14.5)	0.4 (0.0, 0.7)	0.73 (0.57, 0.93)	0.01	
	>1	102/236	82/290	13.4 (4.4, 22.4)	0.7 (0.2, 1.2)	0.65 (0.48, 0.88)	0.003	
Margin	Negative	222/878	244/1182	3.4 (−1.3, 8.2)	0.2 (0.0, 0.4)	0.83 (0.69, 1.00)	0.046	0.48
	Positive	336/1328	310/1619	4.4 (0.8, 7.9)	0.3 (0.2, 0.5)	0.76 (0.65, 0.89)	< 0.001	
SVI	No	403/1811	434/2325	1.7 (−1.3, 4.7)	0.1 (0.0, 0.3)	0.87 (0.75, 0.99)	0.04	0.03
	Yes	150/380	117/438	11.2 (3.3, 19.1)	0.6 (0.3, 1.0)	0.63 (0.49, 0.80)	< 0.001	
ECE	No	314/1221	311/1685	5.9 (2.1, 9.6)	0.3 (0.1, 0.5)	0.76 (0.65, 0.89)	< 0.001	0.34
	Yes	239/931	235/1078	2.1 (−2.4, 6.5)	0.2 (0.0, 0.5)	0.82 (0.68, 0.98)	0.03	

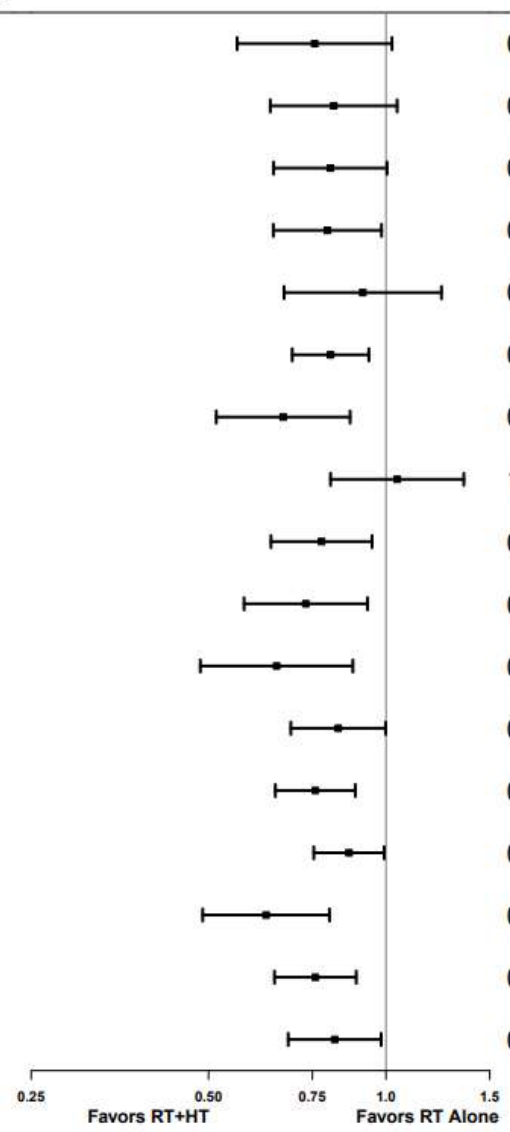

Supplementary Figure 3. Metastasis-Free Survival Impact of Adding Hormonal Therapy to Post-Prostatectomy Radiotherapy Based on Pre-Radiotherapy PSA Strata.

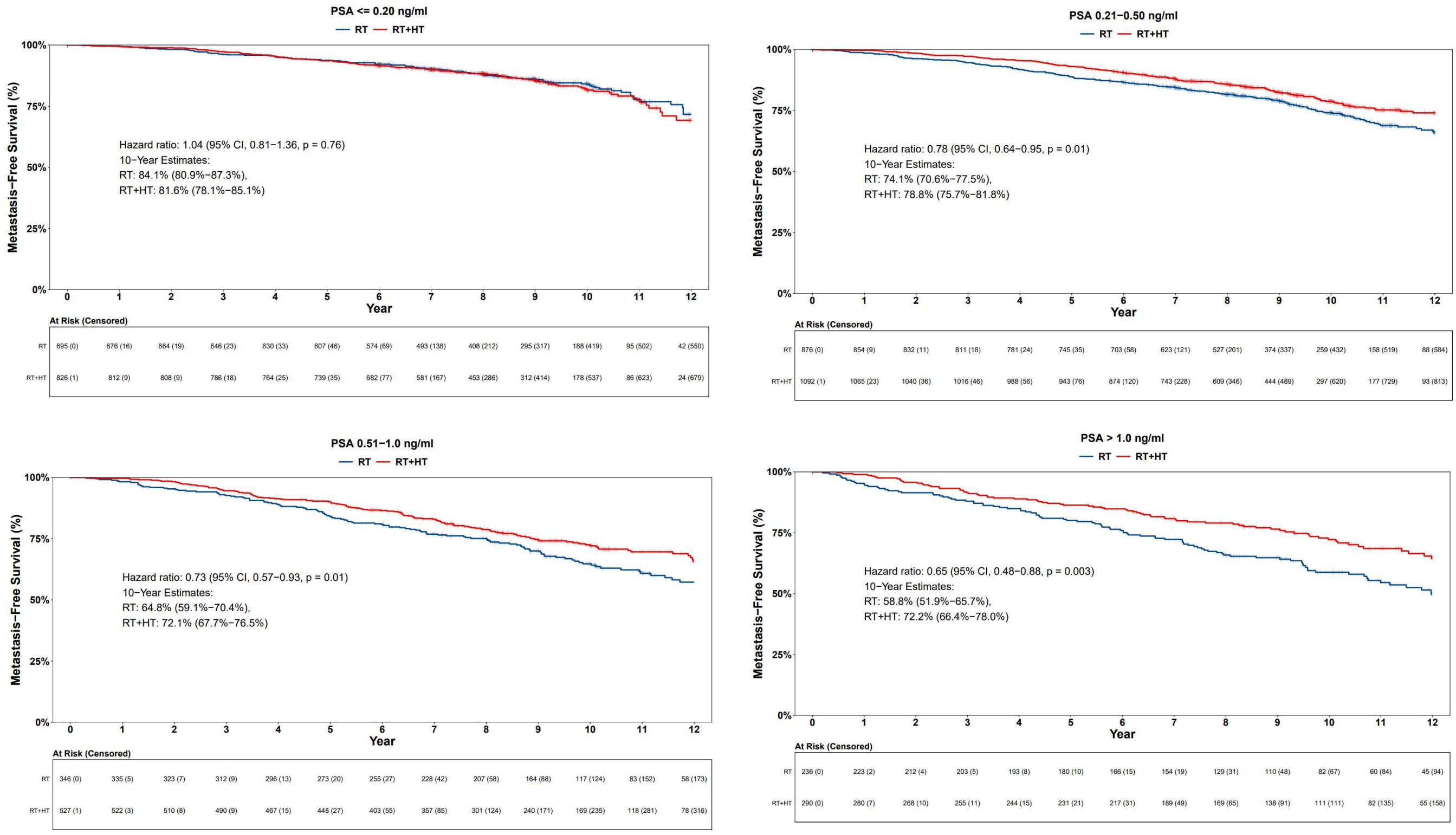

Supplementary Figure 4. Forest plot showing trial-specific effects of short-term hormonal therapy (HT) use on overall survival (OS), metastasis-free survival (MFS), event-free survival (EFS), and biochemical recurrence (BCR). 10-year absolute benefits as well as 10-year restricted mean survival time (RMFST) benefits are shown, along with hazard ratios (HR). 95% confidence intervals (CI 95%) are shown for all estimates, as are p-values for HRs. Outcome incidence is also shown. GETUG, Groupe d'Etude des Tumeurs Uro-Genitales; N, number of at-risk patients; RADICALS, Radiotherapy and Androgen Deprivation In Combination After Local Surgery; RTOG, Radiation Therapy Oncology Group

Endpoint	Study	RT Incidence/N	RT+ST−HT Incidence/N	10−yr Absolute Benefit of RT+ST−HT (%, CI 95%)	10−yr RMST Benefit of RT+ST−HT (Years, CI 95%)	HR (95%)	p value
OS	GETUG−16	51/373	46/369	0.5 (−5.4, 6.4)	0.1 (−0.1, 0.3)	0.94 (0.63, 1.40)	0.75
	RADICALS (2−way)	76/571	69/579	0.4 (−4.6, 5.4)	0.0 (−0.2, 0.2)	0.90 (0.65, 1.25)	0.53
	RADICALS (3−way)	22/166	23/164	−2.9 (−13.0, 7.3)	0.1 (−0.3, 0.4)	1.04 (0.58, 1.87)	0.88
	RTOG−0534	69/564	132/1152	−0.2 (−5.1, 4.7)	0.1 (−0.1, 0.3)	0.91 (0.68, 1.22)	0.55
	Combined	218/1674	270/2264	−0.3 (−3.1, 2.6)	0.1 (−0.1, 0.2)	0.93 (0.77, 1.11)	0.39
MFS	GETUG−16	106/373	77/369	6.6 (−1.0, 14.3)	0.4 (0.1, 0.7)	0.74 (0.55, 0.99)	0.04
	RADICALS (2−way)	110/571	95/579	2.3 (−3.2, 7.9)	0.1 (−0.1, 0.4)	0.85 (0.65, 1.12)	0.26
	RADICALS (3−way)	32/166	31/164	−3.3 (−14.3, 7.6)	0.0 (−0.4, 0.5)	0.96 (0.59, 1.58)	0.89
	RTOG−0534	109/564	189/1152	2.8 (−2.7, 8.3)	0.3 (0.0, 0.5)	0.82 (0.65, 1.03)	0.09
	Combined	357/1674	392/2264	3.2 (−0.1, 6.5)	0.2 (0.1, 0.4)	0.82 (0.71, 0.95)	0.006
EFS	GETUG−16	187/373	120/369	14.9 (7.2, 22.7)	1.4 (0.9, 1.9)	0.56 (0.44, 0.70)	< 0.001
	RADICALS (2−way)	265/571	190/579	14.9 (8.7, 21.1)	1.1 (0.7, 1.5)	0.62 (0.52, 0.75)	< 0.001
	RADICALS (3−way)	78/166	65/164	4.7 (−7.9, 17.3)	0.8 (0.0, 1.5)	0.76 (0.55, 1.06)	0.11
	RTOG−0534	278/564	379/1152	16.5 (10.5, 22.5)	1.4 (1.0, 1.7)	0.56 (0.48, 0.65)	< 0.001
	Combined	808/1674	754/2264	14.1 (10.6, 17.7)	1.3 (1.0, 1.5)	0.59 (0.54, 0.66)	< 0.001
BCR	GETUG−16	124/373	65/369	15.3 (9.0, 21.6)	1.3 (0.8, 1.8)	0.47 (0.35, 0.64)	< 0.001
	RADICALS (2−way)	221/571	142/579	14.9 (9.3, 20.5)	1.2 (0.8, 1.6)	0.56 (0.46, 0.69)	< 0.001
	RADICALS (3−way)	65/166	48/164	10.9 (−0.1, 22.0)	0.9 (0.1, 1.6)	0.69 (0.48, 1.00)	0.047
	RTOG−0534	245/564	301/1152	18.5 (12.7, 24.4)	1.4 (1.0, 1.7)	0.51 (0.43, 0.60)	< 0.001
	Combined	655/1674	556/2264	14.6 (11.4, 17.9)	1.2 (1.0, 1.5)	0.53 (0.47, 0.60)	< 0.001

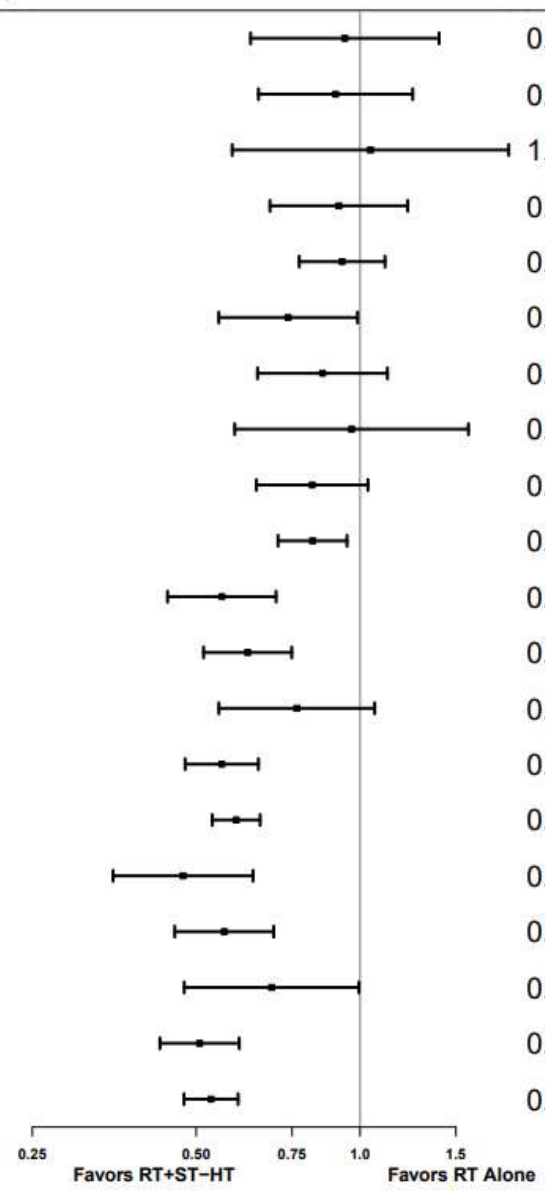

Supplementary Figure 5. Forest plot showing impact of adding short-term hormonal therapy (ST-HT) to post-prostatectomy radiotherapy (RT) on overall survival (top) and metastasis-free survival (bottom), stratified by subgroups of interest. CI, confidence interval; ECE, extracapsular extension; HR, N, number at risk; RMST, restricted mean survival time; SVI, seminal vesicle invasion

Endpoint	Age	RT Incidence/N	RT+ST−HT Incidence/N	10−yr Absolute Benefit of RT+ST−HT (%, CI 95%)	10−yr RMST Benefit of RT+ST−HT (Years, CI 95%)	HR (95%)	p value	Interaction p value
Age	<60	28/345	31/478	0.4 (−4.2, 4.9)	0.0 (−0.2, 0.2)	0.89 (0.52, 1.51)	0.63	0.54
	60−64	38/415	62/554	−3.0 (−8.0, 1.9)	0.0 (−0.2, 0.2)	1.22 (0.81, 1.84)	0.35	
	65−69	66/494	85/691	−0.6 (−6.0, 4.8)	0.1 (−0.1, 0.3)	0.92 (0.67, 1.28)	0.62	
	70+	86/420	92/541	1.9 (−4.9, 8.7)	0.1 (−0.2, 0.4)	0.82 (0.61, 1.11)	0.24	
Gleason	6 or lower	36/270	38/384	3.3 (−2.7, 9.2)	0.1 (−0.2, 0.4)	0.79 (0.50, 1.26)	0.30	0.65
	7	141/1188	179/1562	−1.1 (−4.4, 2.2)	0.0 (−0.1, 0.2)	0.99 (0.79, 1.24)	0.92	
	8−10	41/216	53/318	−1.3 (−11.6, 9.0)	0.2 (−0.2, 0.5)	0.80 (0.53, 1.21)	0.31	
PSA (ng/ml)	<= 0.20	57/598	70/733	−1.9 (−6.1, 2.3)	0.0 (−0.2, 0.2)	1.03 (0.73, 1.47)	0.84	0.56
	0.21−0.50	90/689	112/915	−1.8 (−6.2, 2.7)	0.1 (−0.1, 0.3)	0.96 (0.72, 1.27)	0.86	
	0.51−1	42/221	59/378	5.5 (−3.3, 14.4)	0.0 (−0.3, 0.4)	0.82 (0.55, 1.23)	0.34	
	>1	25/122	25/182	8.1 (−3.4, 19.6)	0.2 (−0.2, 0.7)	0.69 (0.39, 1.23)	0.15	
Margin	Negative	95/726	122/1030	−0.2 (−4.5, 4.1)	0.0 (−0.2, 0.2)	0.89 (0.67, 1.16)	0.39	0.91
	Positive	122/938	147/1225	−0.1 (−4.0, 3.7)	0.1 (0.0, 0.3)	0.95 (0.74, 1.21)	0.65	
SVI	No	171/1406	218/1888	−0.9 (−3.9, 2.1)	0.0 (−0.1, 0.1)	0.99 (0.81, 1.21)	0.88	0.38
	Yes	44/250	51/341	1.7 (−6.7, 10.2)	0.3 (0.0, 0.6)	0.77 (0.51, 1.17)	0.23	
ECE	No	123/924	144/1387	1.3 (−2.5, 5.0)	0.1 (0.0, 0.3)	0.78 (0.61, 1.00)	0.04	0.06
	Yes	92/697	120/837	−2.0 (−6.6, 2.5)	0.0 (−0.2, 0.2)	1.09 (0.83, 1.44)	0.50	

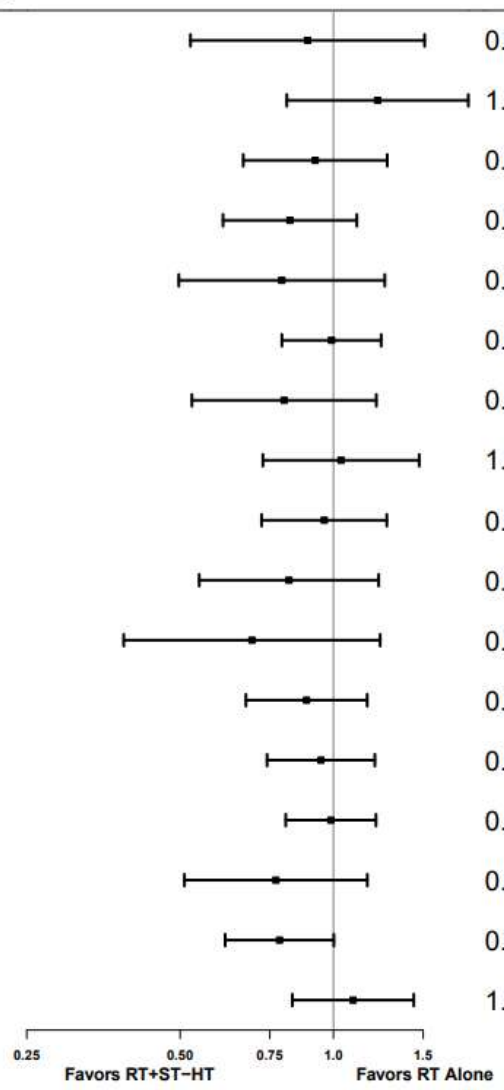

Endpoint	Age	RT Incidence/N	RT+ST−HT Incidence/N	10−yr Absolute Benefit of RT+ST−HT (%, CI 95%)	10−yr RMST Benefit of RT+ST−HT (Years, CI 95%)	HR (95%)	p value	Interaction p value
Age	<60	60/345	59/478	6.4 (−0.1, 12.9)	0.2 (−0.1, 0.5)	0.78 (0.54, 1.14)	0.18	0.37
	60−64	75/415	102/554	−2.2 (−8.4, 3.9)	0.0 (−0.3, 0.3)	1.01 (0.74, 1.37)	0.95	
	65−69	102/494	116/691	1.0 (−5.0, 7.1)	0.2 (0.0, 0.5)	0.81 (0.62, 1.06)	0.12	
	70+	120/420	115/541	8.2 (0.7, 15.7)	0.4 (0.0, 0.7)	0.73 (0.56, 0.94)	0.02	
Gleason	6 or lower	40/270	50/384	1.3 (−5.2, 7.7)	0.1 (−0.2, 0.4)	0.93 (0.61, 1.42)	0.74	0.71
	7	244/1188	258/1562	3.4 (−0.6, 7.4)	0.2 (0.0, 0.3)	0.83 (0.69, 0.99)	0.03	
	8−10	73/216	84/318	4.6 (−6.5, 15.8)	0.6 (0.1, 1.1)	0.71 (0.52, 0.98)	0.03	
PSA (ng/ml)	<= 0.20	87/598	105/733	−2.6 (−7.8, 2.6)	0.0 (−0.2, 0.2)	1.05 (0.79, 1.40)	0.76	0.29
	0.21−0.50	157/689	154/915	4.2 (−1.1, 9.6)	0.4 (0.1, 0.6)	0.75 (0.60, 0.94)	0.01	
	0.51−1	69/221	89/378	10.7 (1.3, 20.1)	0.3 (−0.1, 0.8)	0.73 (0.53, 1.01)	0.06	
	>1	39/122	40/182	14.6 (1.7, 27.5)	0.5 (−0.2, 1.2)	0.76 (0.48, 1.20)	0.16	
Margin	Negative	162/726	193/1030	2.6 (−2.5, 7.8)	0.1 (−0.1, 0.4)	0.85 (0.69, 1.05)	0.12	0.61
	Positive	194/938	198/1225	3.9 (−0.5, 8.3)	0.3 (0.1, 0.5)	0.78 (0.64, 0.95)	0.02	
SVI	No	274/1406	310/1888	2.1 (−1.5, 5.6)	0.1 (0.0, 0.3)	0.87 (0.74, 1.02)	0.09	0.24
	Yes	79/250	81/341	7.3 (−2.1, 16.8)	0.5 (0.1, 1.0)	0.69 (0.50, 0.94)	0.02	
ECE	No	194/924	214/1387	5.5 (1.1, 9.8)	0.3 (0.1, 0.5)	0.74 (0.61, 0.90)	0.002	0.13
	Yes	158/697	171/837	0.7 (−4.7, 6.2)	0.2 (−0.1, 0.4)	0.90 (0.72, 1.12)	0.36	

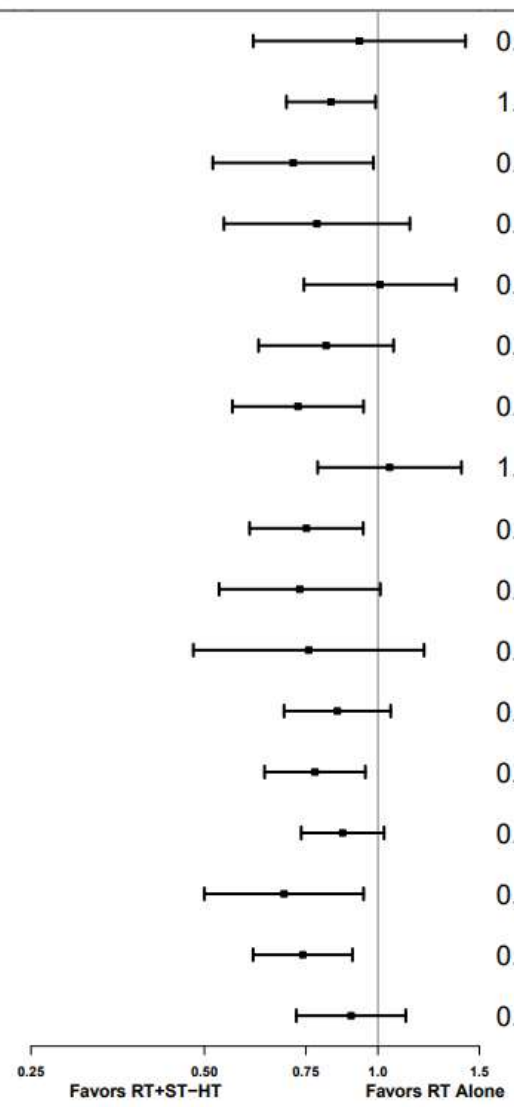

Supplementary Figure 6. Forest plot showing trial-specific effects of long-term hormonal therapy (HT) use on overall survival (OS), metastasis-free survival (MFS), event-free survival (EFS), and biochemical recurrence (BCR). 10-year absolute benefits as well as 10-year restricted mean survival time (RMFST) benefits are shown, along with hazard ratios (HR). 95% confidence intervals (CI 95%) are shown for all estimates, as are p-values for HRs. Outcome incidence is also shown. N, number of at-risk patients; RADICALS, Radiotherapy and Androgen Deprivation In Combination After Local Surgery; RTOG, Radiation Therapy Oncology Group

Endpoint	Study	RT Incidence/N	RT+LT−HT Incidence/N	10−yr Absolute Benefit of RT+LT−HT (%, CI 95%)	10−yr RMST Benefit of RT+LT−HT (Years, CI 95%)	HR (95%)	p value
OS	RADICALS (3−way)	22/166	21/162	−0.4 (−9.7, 9.0)	0.0 (−0.4, 0.4)	0.94 (0.52, 1.71)	0.85
	RTOG−9601	131/376	108/384	4.0 (−1.9, 9.8)	0.3 (0.0, 0.5)	0.77 (0.59, 0.99)	0.04
	Combined	153/542	129/546	2.9 (−2.1, 7.8)	0.2 (0.0, 0.4)	0.79 (0.63, 1.00)	0.049
MFS	RADICALS (3−way)	32/166	26/162	0.6 (−9.8, 11.0)	0.1 (−0.4, 0.5)	0.80 (0.48, 1.34)	0.40
	RTOG−9601	170/376	137/384	7.3 (0.7, 13.9)	0.5 (0.2, 0.9)	0.73 (0.58, 0.92)	0.006
	Combined	202/542	163/546	5.6 (0.1, 11.2)	0.4 (0.1, 0.7)	0.74 (0.60, 0.91)	0.004
EFS	RADICALS (3−way)	78/166	41/162	20.9 (9.3, 32.6)	1.8 (1.1, 2.5)	0.43 (0.30, 0.63)	< 0.001
	RTOG−9601	288/376	237/384	16.8 (9.9, 23.8)	1.6 (1.2, 2.1)	0.60 (0.50, 0.71)	< 0.001
	Combined	366/542	278/546	18.2 (12.1, 24.4)	1.7 (1.3, 2.1)	0.56 (0.48, 0.66)	< 0.001
BCR	RADICALS (3−way)	65/166	27/162	23.7 (13.5, 34.0)	2.0 (1.3, 2.6)	0.34 (0.22, 0.53)	< 0.001
	RTOG−9601	252/376	169/384	24.3 (17.2, 31.3)	2.0 (1.5, 2.5)	0.48 (0.40, 0.58)	< 0.001
	Combined	317/542	196/546	24.3 (18.2, 30.3)	2.0 (1.6, 2.4)	0.46 (0.38, 0.54)	< 0.001

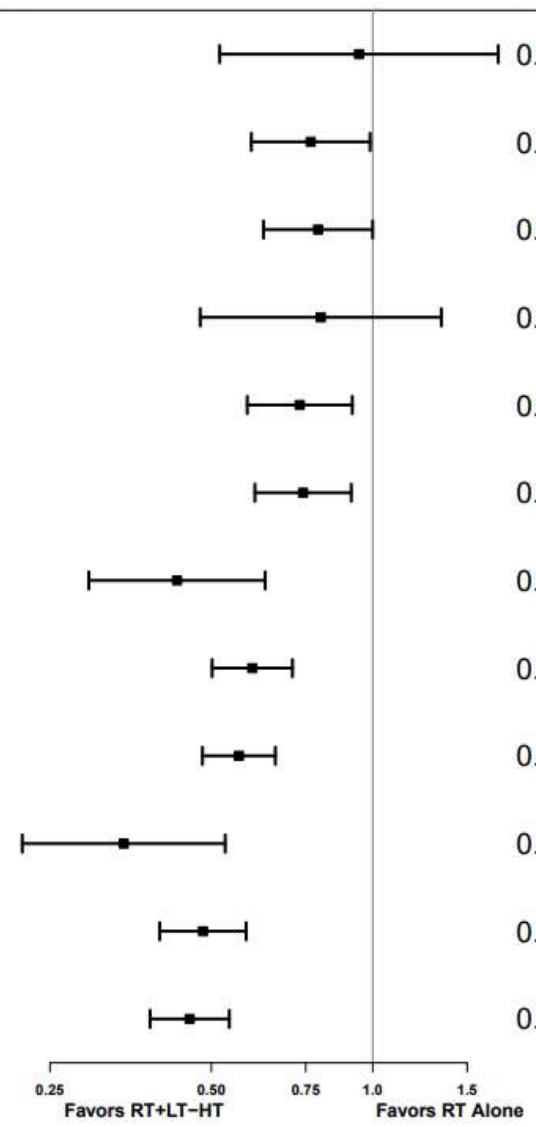

Supplementary Figure 7. Forest plot showing impact of adding long-term hormonal therapy (LT-HT) to post-prostatectomy radiotherapy (RT) on overall survival (top) and metastasis-free survival (bottom), stratified by subgroups of interest. CI, confidence interval; ECE, extracapsular extension; HR, N, number at risk; RMST, restricted mean survival time; SVI, seminal vesicle invasion

Endpoint	Age	RT Incidence/N	RT+LT−HT Incidence/N	10−yr Absolute Benefit of RT+LT−HT (%, CI 95%)	10−yr RMST Benefit of RT+LT−HT (Years, CI 95%)	HR (95%)	p value	Interaction p value
Age	<60	20/123	12/123	7.4 (0.1, 14.7)	0.2 (−0.2, 0.6)	0.52 (0.25, 1.09)	0.08	0.44
	60−64	38/147	23/123	9.6 (1.6, 17.6)	0.4 (0.1, 0.8)	0.61 (0.36, 1.02)	0.048	
	65−69	40/144	42/167	−2.5 (−12.0, 6.9)	0.2 (−0.2, 0.7)	0.85 (0.55, 1.31)	0.42	
	70+	55/128	52/133	−0.9 (−13.4, 11.6)	−0.1 (−0.7, 0.5)	0.97 (0.66, 1.42)	0.90	
Gleason	6 or lower	32/120	35/126	−1.7 (−11.4, 8.1)	0.0 (−0.4, 0.5)	1.09 (0.68, 1.77)	0.74	0.37
	7	82/319	66/320	3.9 (−2.5, 10.2)	0.2 (−0.1, 0.5)	0.75 (0.54, 1.04)	0.09	
	8−10	35/89	27/86	2.8 (−11.0, 16.5)	0.1 (−0.6, 0.8)	0.70 (0.42, 1.15)	0.14	
PSA (ng/ml)	<= 0.20	12/97	17/93	−6.6 (−17.6, 4.5)	−0.3 (−0.8, 0.2)	1.74 (0.82, 3.66)	0.15	0.12
	0.21−0.50	43/187	35/177	2.2 (−5.8, 10.3)	0.1 (−0.2, 0.4)	0.89 (0.57, 1.40)	0.63	
	0.51−1	50/125	41/149	6.1 (−3.7, 15.9)	0.5 (0.0, 1.0)	0.63 (0.41, 0.95)	0.02	
	>1	47/114	34/108	7.8 (−4.3, 19.8)	0.4 (−0.2, 1.0)	0.69 (0.44, 1.07)	0.09	
Margin	Negative	40/152	38/152	−1.3 (−11.0, 8.4)	0.0 (−0.3, 0.4)	0.92 (0.59, 1.43)	0.72	0.40
	Positive	113/390	91/394	4.4 (−1.3, 10.2)	0.2 (0.0, 0.5)	0.74 (0.56, 0.98)	0.03	
SVI	No	104/405	98/437	0.6 (−4.7, 5.9)	0.1 (−0.2, 0.3)	0.85 (0.64, 1.12)	0.25	0.52
	Yes	48/130	30/97	7.5 (−5.2, 20.1)	0.4 (−0.2, 1.0)	0.72 (0.46, 1.14)	0.16	
ECE	No	89/297	81/298	−0.8 (−7.7, 6.2)	0.0 (−0.4, 0.3)	0.95 (0.70, 1.28)	0.70	0.06
	Yes	64/234	47/241	8.2 (1.1, 15.3)	0.5 (0.2, 0.8)	0.58 (0.39, 0.85)	0.004	

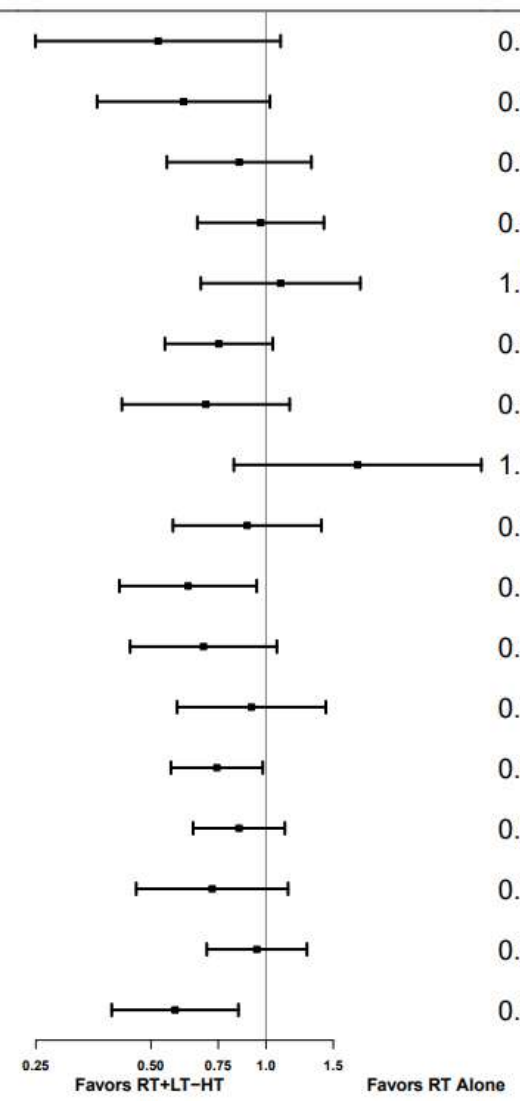

Endpoint	Age	RT Incidence/N	RT+LT−HT Incidence/N	10−yr Absolute Benefit of RT+LT−HT (%, CI 95%)	10−yr RMST Benefit of RT+LT−HT (Years, CI 95%)	HR (95%)	p value	Interaction p value
Age	<60	33/123	26/123	5.3 (−5.2, 15.8)	0.4 (−0.1, 0.9)	0.71 (0.42, 1.20)	0.20	0.25
	60−64	54/147	28/123	16.6 (6.8, 26.4)	0.8 (0.2, 1.3)	0.51 (0.32, 0.81)	0.003	
	65−69	52/144	50/167	0.1 (−10.1, 10.3)	0.4 (−0.1, 0.9)	0.79 (0.54, 1.17)	0.21	
	70+	63/128	59/133	1.2 (−11.9, 14.2)	0.0 (−0.6, 0.7)	0.94 (0.66, 1.34)	0.77	
Gleason	6 or lower	39/120	39/126	3.8 (−7.5, 15.1)	0.3 (−0.3, 0.8)	0.91 (0.58, 1.42)	0.67	0.37
	7	112/319	90/320	5.9 (−1.2, 13.0)	0.3 (0.0, 0.7)	0.75 (0.57, 0.99)	0.04	
	8−10	46/89	31/86	7.0 (−7.9, 22.0)	0.8 (−0.1, 1.6)	0.59 (0.38, 0.94)	0.02	
PSA (ng/ml)	<= 0.20	22/97	19/93	−1.9 (−14.2, 10.4)	−0.1 (−0.7, 0.5)	1.03 (0.56, 1.91)	0.95	0.38
	0.21−0.50	55/187	44/177	5.1 (−4.0, 14.2)	0.2 (−0.2, 0.6)	0.86 (0.58, 1.28)	0.47	
	0.51−1	60/125	56/149	5.6 (−5.6, 16.8)	0.6 (0.1, 1.2)	0.73 (0.51, 1.05)	0.08	
	>1	63/114	42/108	13.9 (1.0, 26.9)	0.9 (0.1, 1.7)	0.59 (0.40, 0.87)	0.007	
Margin	Negative	60/152	51/152	6.2 (−5.1, 17.6)	0.3 (−0.2, 0.8)	0.78 (0.53, 1.13)	0.18	0.78
	Positive	142/390	112/394	5.4 (−1.0, 11.8)	0.4 (0.1, 0.7)	0.73 (0.57, 0.94)	0.01	
SVI	No	129/405	124/437	1.5 (−4.5, 7.4)	0.2 (−0.1, 0.5)	0.86 (0.67, 1.10)	0.24	0.050
	Yes	71/130	36/97	16.1 (2.4, 29.8)	0.8 (0.2, 1.5)	0.55 (0.37, 0.82)	0.003	
ECE	No	120/297	97/298	5.4 (−2.3, 13.2)	0.3 (−0.1, 0.7)	0.79 (0.61, 1.04)	0.09	0.46
	Yes	81/234	64/241	7.0 (−1.1, 15.1)	0.6 (0.2, 1.0)	0.66 (0.48, 0.92)	0.01	

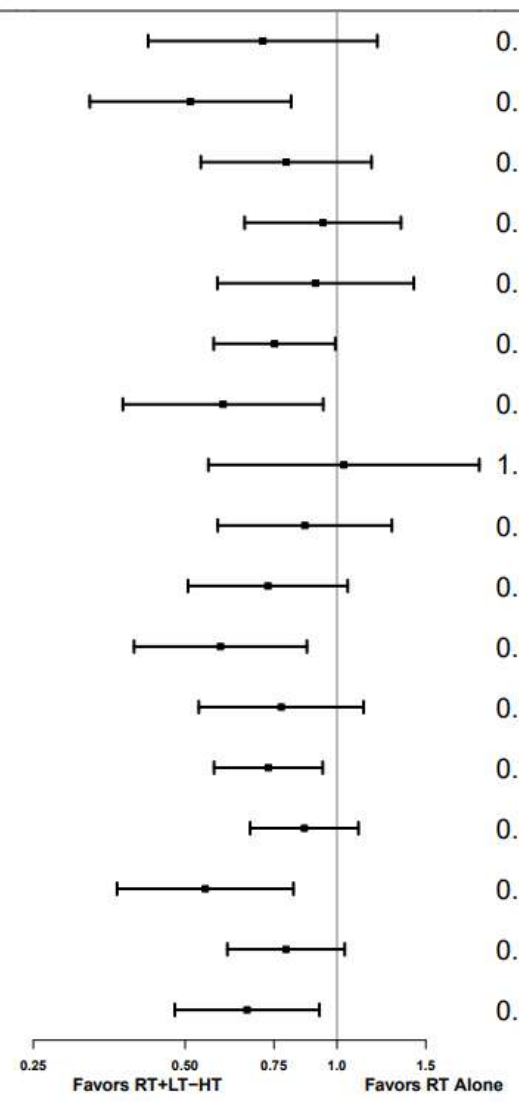

Supplementary Figure 8. Forest plot showing trial-specific effects of long-term hormonal therapy (HT) use *versus* short-term hormonal therapy use on overall survival (OS), metastasis-free survival (MFS), event-free survival (EFS), and biochemical recurrence (BCR). 10-year absolute benefits as well as 10-year restricted mean survival time (RMFST) benefits are shown, along with hazard ratios (HR). 95% confidence intervals (CI 95%) are shown for all estimates, as are p-values for HRs. Outcome incidence is also shown. N, number of at-risk patients; RADICALS, Radiotherapy and Androgen Deprivation In Combination After Local Surgery

Endpoint	Study	RT+ST−HT Incidence/N	RT+LT−HT Incidence/N	10−yr Absolute Benefit of RT+LT−HT (%, CI 95%)	10−yr RMST Benefit of RT+LT−HT (Years, CI 95%)	HR (95%)	p value
OS	RADICALS (2−way)	88/597	79/600	2.5 (−2.8, 7.8)	0.1 (−0.1, 0.3)	0.88 (0.65, 1.20)	0.43
	RADICALS (3−way)	23/164	21/162	2.5 (−7.8, 12.7)	0.0 (−0.4, 0.3)	0.89 (0.49, 1.61)	0.70
	Combined	111/761	100/762	2.5 (−2.2, 7.3)	0.1 (−0.1, 0.3)	0.89 (0.68, 1.16)	0.37
MFS	RADICALS (2−way)	143/597	113/600	6.9 (0.7, 13.1)	0.4 (0.1, 0.6)	0.75 (0.59, 0.96)	0.02
	RADICALS (3−way)	31/164	26/162	3.9 (−7.3, 15.0)	0.1 (−0.4, 0.5)	0.82 (0.49, 1.38)	0.46
	Combined	174/761	139/762	6.2 (0.8, 11.6)	0.3 (0.1, 0.5)	0.76 (0.61, 0.95)	0.02
EFS	RADICALS (2−way)	279/597	225/600	8.0 (1.7, 14.3)	1.0 (0.6, 1.4)	0.70 (0.59, 0.83)	< 0.001
	RADICALS (3−way)	65/164	41/162	16.2 (4.0, 28.5)	1.0 (0.4, 1.7)	0.56 (0.38, 0.83)	0.003
	Combined	344/761	266/762	9.8 (4.2, 15.4)	1.0 (0.7, 1.4)	0.67 (0.57, 0.79)	< 0.001
BCR	RADICALS (2−way)	236/597	175/600	9.4 (3.7, 15.2)	1.1 (0.7, 1.5)	0.64 (0.53, 0.77)	< 0.001
	RADICALS (3−way)	48/164	27/162	12.8 (3.0, 22.7)	1.1 (0.5, 1.7)	0.50 (0.32, 0.80)	0.004
	Combined	284/761	202/762	10.2 (5.2, 15.2)	1.1 (0.8, 1.4)	0.62 (0.52, 0.73)	< 0.001

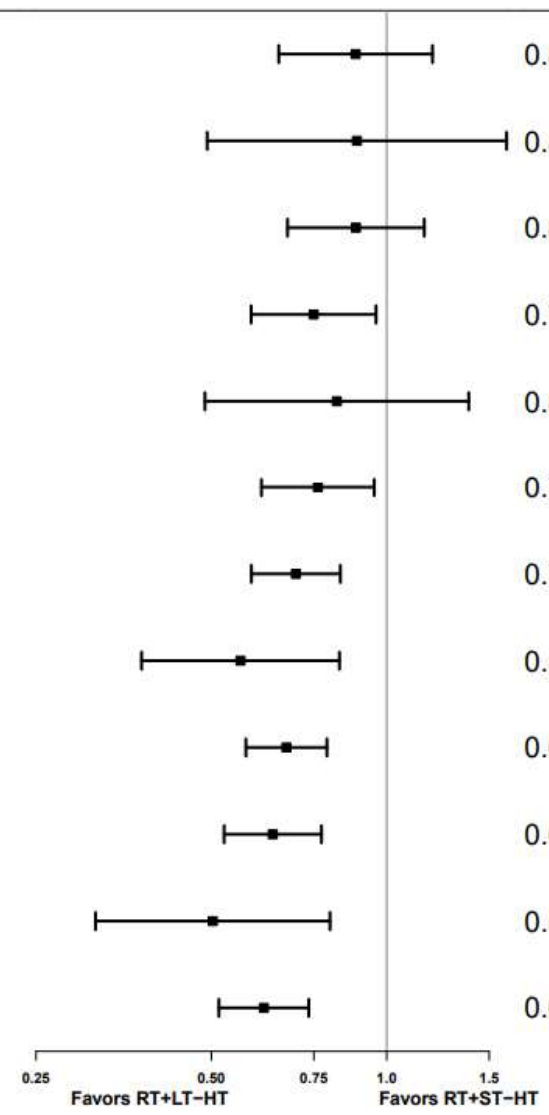

Supplementary Figure 9. Forest plot showing impact of prolonging short-term hormonal therapy (ST-HT) to long-term hormonal therapy (LT-HT) with post-prostatectomy radiotherapy (RT) on overall survival (top) and metastasis-free survival (bottom), stratified by subgroups of interest. CI, confidence interval; ECE, extracapsular extension; HR, N, number at risk; RMST, restricted mean survival time; SVI, seminal vesicle invasion

Endpoint	Age	RT+ST−HT Incidence/N	RT+LT−HT Incidence/N	10−yr Absolute Benefit of RT+LT−HT (%, CI 95%)	10−yr RMST Benefit of RT+LT−HT (Years, CI 95%)	HR (95%)	p value	Interaction p value
Age	<60	17/171	17/155	−4.0 (−13.1, 5.2)	0.0 (−0.3, 0.4)	1.07 (0.55, 2.10)	0.84	0.006
	60−64	30/182	10/188	14.8 (6.7, 22.9)	0.6 (0.2, 0.9)	0.31 (0.15, 0.63)	< 0.001	
	65−69	40/262	37/248	3.6 (−4.7, 11.8)	0.0 (−0.3, 0.3)	0.97 (0.62, 1.51)	0.87	
	70+	24/146	36/171	−6.4 (−19.1, 6.3)	−0.2 (−0.6, 0.2)	1.37 (0.81, 2.34)	0.25	
Gleason	6 or lower	7/61	11/53	−16.1 (−34.6, 2.3)	−0.6 (−1.2, −0.1)	2.19 (0.84, 5.66)	0.10	0.07
	7	66/484	49/490	5.0 (−0.6, 10.6)	0.3 (0.1, 0.5)	0.71 (0.49, 1.03)	0.06	
	8−10	38/215	40/219	2.2 (−7.1, 11.6)	−0.1 (−0.4, 0.3)	1.02 (0.65, 1.59)	0.92	
PSA (ng/ml)	<= 0.20	43/350	38/341	0.8 (−6.2, 7.9)	0.1 (−0.2, 0.3)	0.93 (0.60, 1.44)	0.74	0.92
	0.21−0.50	36/207	28/211	5.3 (−4.1, 14.7)	0.2 (−0.1, 0.5)	0.75 (0.46, 1.23)	0.24	
	0.51−1	10/82	11/89	−0.7 (−13.4, 12.1)	0.1 (−0.4, 0.5)	0.97 (0.41, 2.28)	1.00	
	>1	18/86	18/86	4.0 (−9.4, 17.5)	0.2 (−0.4, 0.8)	0.79 (0.40, 1.54)	0.63	
Margin	Negative	42/281	36/278	1.8 (−6.4, 10.0)	0.1 (−0.2, 0.4)	0.88 (0.56, 1.37)	0.55	0.94
	Positive	69/480	64/484	2.9 (−2.9, 8.7)	0.1 (−0.1, 0.3)	0.89 (0.64, 1.26)	0.51	
SVI	No	73/509	53/516	6.0 (0.4, 11.7)	0.2 (0.0, 0.4)	0.69 (0.48, 0.98)	0.04	0.02
	Yes	36/230	44/220	−4.7 (−14.1, 4.7)	−0.1 (−0.4, 0.2)	1.30 (0.83, 2.03)	0.24	
ECE	No	27/229	30/237	−0.9 (−9.3, 7.4)	−0.1 (−0.4, 0.2)	1.10 (0.65, 1.85)	0.71	0.25
	Yes	82/497	65/497	5.0 (−1.0, 10.9)	0.2 (0.0, 0.5)	0.76 (0.55, 1.06)	0.10	
Pre−RP PSA (ng/ml)	<= 9	55/339	44/343	6.7 (−0.5, 13.8)	0.1 (−0.1, 0.4)	0.80 (0.53, 1.18)	0.23	0.55
	> 9	48/370	46/365	0.3 (−6.6, 7.2)	0.1 (−0.1, 0.4)	0.92 (0.62, 1.39)	0.70	

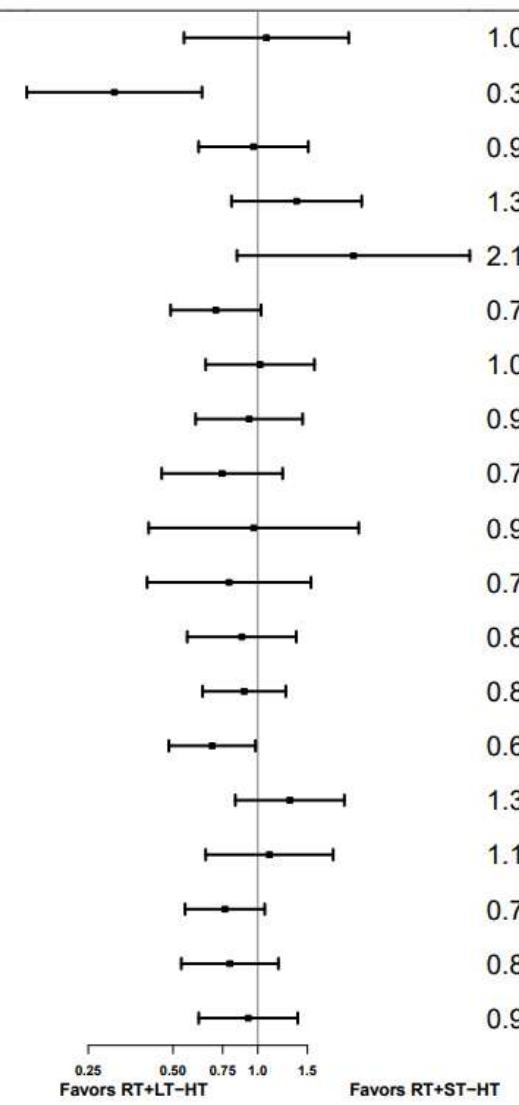

Endpoint	Age	RT+ST−HT Incidence/N	RT+LT−HT Incidence/N	10−yr Absolute Benefit of RT+LT−HT (%, CI 95%)	10−yr RMST Benefit of RT+LT−HT (Years, CI 95%)	HR (95%)	p value	Interaction p value
Age	<60	33/171	29/155	1.2 (−10.6, 13.0)	0.0 (−0.5, 0.5)	0.93 (0.56, 1.53)	0.77	0.04
	60−64	44/182	21/188	15.8 (6.0, 25.5)	0.7 (0.3, 1.1)	0.44 (0.26, 0.74)	0.001	
	65−69	62/262	44/248	11.8 (2.8, 20.8)	0.4 (0.0, 0.8)	0.71 (0.48, 1.04)	0.07	
	70+	35/146	45/171	−9.4 (−23.1, 4.4)	0.0 (−0.5, 0.5)	1.14 (0.73, 1.79)	0.61	
Gleason	6 or lower	7/61	11/53	−14.0 (−32.8, 4.7)	−0.6 (−1.2, 0.0)	2.16 (0.84, 5.61)	0.11	0.03
	7	102/484	68/490	10.0 (3.3, 16.7)	0.5 (0.2, 0.7)	0.62 (0.45, 0.84)	0.002	
	8−10	64/215	60/219	3.7 (−6.6, 14.0)	0.2 (−0.3, 0.7)	0.86 (0.61, 1.23)	0.42	
PSA (ng/ml)	<= 0.20	65/350	46/341	3.6 (−3.7, 11.0)	0.3 (0.0, 0.6)	0.71 (0.49, 1.04)	0.07	0.93
	0.21−0.50	50/207	42/211	6.2 (−5.0, 17.4)	0.3 (−0.1, 0.7)	0.79 (0.52, 1.19)	0.25	
	0.51−1	19/82	18/89	6.1 (−9.9, 22.2)	0.1 (−0.5, 0.8)	0.85 (0.45, 1.62)	0.61	
	>1	35/86	27/86	15.4 (−2.2, 32.9)	0.8 (0.0, 1.7)	0.63 (0.38, 1.05)	0.12	
Margin	Negative	68/281	54/278	5.3 (−4.6, 15.3)	0.2 (−0.1, 0.6)	0.80 (0.56, 1.14)	0.19	0.84
	Positive	106/480	85/484	6.6 (0.1, 13.1)	0.3 (0.1, 0.6)	0.75 (0.56, 0.99)	0.047	
SVI	No	106/509	74/516	7.9 (1.7, 14.1)	0.4 (0.1, 0.6)	0.66 (0.49, 0.89)	0.006	0.13
	Yes	64/230	61/220	2.7 (−8.8, 14.1)	0.2 (−0.2, 0.7)	0.92 (0.65, 1.31)	0.62	
ECE	No	38/229	39/237	2.3 (−7.3, 11.8)	0.0 (−0.3, 0.4)	0.98 (0.63, 1.53)	0.96	0.11
	Yes	134/497	93/497	9.6 (2.7, 16.5)	0.5 (0.2, 0.8)	0.65 (0.50, 0.84)	< 0.001	
Pre−RP PSA (ng/ml)	<= 9	86/339	63/343	9.9 (0.9, 18.9)	0.4 (0.1, 0.8)	0.69 (0.50, 0.95)	0.02	0.55
	> 9	78/370	64/365	6.0 (−1.7, 13.7)	0.3 (0.0, 0.6)	0.78 (0.56, 1.09)	0.15	

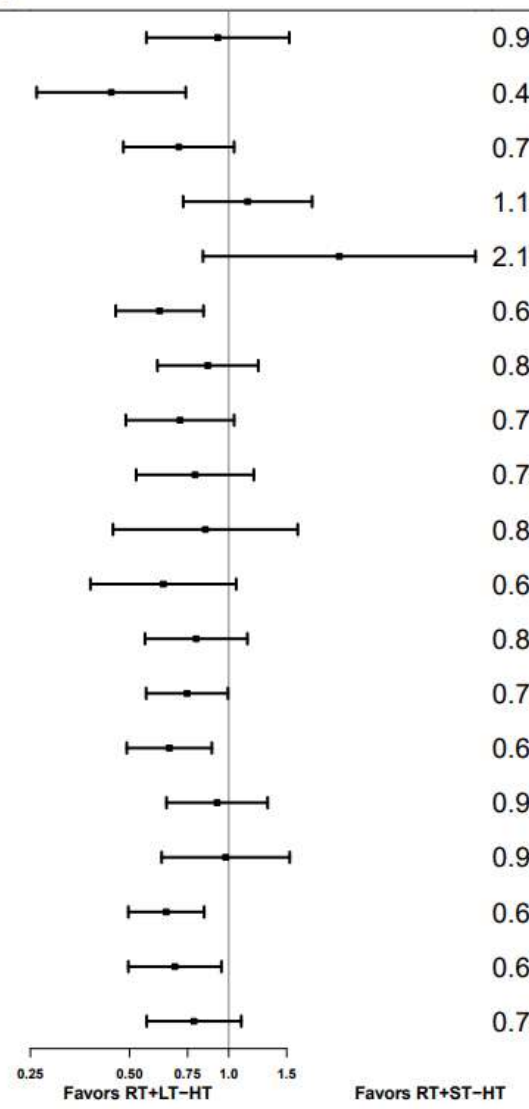

Reference

1 Carrie C, Magné N, Burban-Provost P, *et al.* Short-term androgen deprivation therapy combined with radiotherapy as salvage treatment after radical prostatectomy for prostate cancer (GETUG-AFU 16): a 112-month follow-up of a phase 3, randomised trial. *Lancet Oncol* 2019; **20**: 1740–9.

2 Parker CC, Clarke NW, Cook AD, *et al.* Adding 6 months of androgen deprivation therapy to postoperative radiotherapy for prostate cancer: a comparison of short-course versus no androgen deprivation therapy in the RADICALS-HD randomised controlled trial. *The Lancet* 2024; **403**: 2405–15.

3 Parker CC, Clarke NW, Cook AD, *et al.* Randomised Trial of No, Short-term, or Long-term Androgen Deprivation Therapy with Postoperative Radiotherapy After Radical Prostatectomy: Results from the Three-way Comparison of RADICALS-HD (NCT00541047). *European Urology* 2024; **86**: 422–30.

4 Pollack A, Karrison TG, Balogh AG, *et al.* The addition of androgen deprivation therapy and pelvic lymph node treatment to prostate bed salvage radiotherapy (NRG Oncology/RTOG 0534 SPPORT): an international, multicentre, randomised phase 3 trial. *The Lancet* 2022; **399**: 1886–901.

5 Shipley WU, Seiferheld W, Lukka HR, *et al.* Radiation with or without Antiandrogen Therapy in Recurrent Prostate Cancer. *New England Journal of Medicine* 2017; **376**: 417–28.

6 Parker CC, Kynaston H, Cook AD, *et al.* Duration of androgen deprivation therapy with postoperative radiotherapy for prostate cancer: a comparison of long-course versus short-course androgen deprivation therapy in the RADICALS-HD randomised trial. *The Lancet* 2024; **403**: 2416–25.
